# Diversity of the strongly rheophilous tadpoles of Malagasy tree frogs, genus *Boophis* (Anura, Mantellidae), and identification of new candidate species via larval DNA sequence and morphology

**DOI:** 10.3897/zookeys.178.1410

**Published:** 2012-03-29

**Authors:** Roger Daniel Randrianiaina, Axel Strauß, Julian Glos, Miguel Vences

**Affiliations:** 1 Division of Evolutionary Biology, Zoological Institute, Technical University of Braunschweig, Mendelssohnsstr. 4, 38106 Braunschweig, Germany; 2 Département de Biologie Animale, B.P 906, Université d'Antananarivo, Antananarivo, Madagascar; 3 Institut for Genetik, Ludwig-Maximilians-Universität München, Großhaderner Str. 2-4, 82152 Martinsried, Germany; 4 Zoological Institute, University of Hamburg, Martin-Luther-King Platz 3, 20146 Hamburg, Germany

**Keywords:** Amphibia, Anura, Mantellidae, *Boophis*, larval morphology, oral disc, clasping, adherent, suctorial, candidate species, larval ecology, microhabitat preference

## Abstract

This study provides detailed morphological descriptions of previously unknown tadpoles of the treefrog genus *Boophis* Tschudi and analyses of habitat preferences of several of these tadpoles in Ranomafana National Park. A total of twenty-two tadpoles determined via DNA barcoding are characterized morphologically herein, fourteen of them for the first time. Twelve of these tadpoles belong to taxonomically undescribed candidate species which in several cases are so far only known from their larval stages. Our data show that the larvae of some of these candidate species occur syntopically yet maintaining a clearly correlated genetic and morphological identity, suggesting that they indeed are true biological and evolutionary species. Tadpoles considered to belong to the “adherent” ecomorphological guild inhabit fast-running waters and their oral disc is commonly to continuously attached to the rocky substrate, supposedly to keep their position in the water current. Some of these species are characterized by the presence of a dorsal gap of papillae and the absence of an upper jaw sheath. This guild includes the tadpoles of the *Boophis albipuncatus* group (*Boophis ankaratra*, *Boophis schuboeae*, *Boophis albipunctatus*, *Boophis sibilans*, *Boophis luciae*), and of the *Boophis mandraka* group (*Boophis sambirano* and six candidate species related to this species and to *Boophis mandraka*). Tadpoles considered belonging to the “suctorial” guild inhabit fast-running waters where they use frequently their oral disc to attach to the substrate. They have an enlarged oral disc without any dorsal gap, including two nominal species (*Boophis marojezensis*, *Boophis vittatus*), and five candidate species related to *Boophis marojezensis*. An ecological analysis of the tadpoles of *Boophis luciae*, *Boophis schuboeae* and *Boophis marojezensis* [Ca51 JQ518198] from Ranomafana National Park did not provide evidence for a clear preference of these tadpoles to the fast flowing microhabitat sections of the stream, although the tadpoles discussed in this study are typically caught in this habitat.

## Introduction

The genus *Boophis* Tschudi, 1938 is a species-rich group of treefrogs in the family Mantellidae which is endemic to Madagascar and to the Comoran island of Mayotte. Seventy-two nominal species and over 25 candidate species of *Boophis* are currently known (Vieites et al. 2009, Vallan et al. 2010, Glaw et al. 2010, Vences et al. 2010a, b).

Tadpoles have been described for 46 species of *Boophis* (e.g., Blommers-Schlösser 1979, Thomas et al. 2005, Grosjean et al. 2006, Raharivololoniaina et al. 2006, Altig and McDiarmid 2006, Glos et al. 2007, Schmidt et al. 2008, Randrianiaina et al. 2009a, b, Rasolonjatovo et al. 2010). Compared to many other Malagasy anuran groups, the larval stages of *Boophis* are therefore quite well known, possibly because they are relatively easy to find in rainforest streams (e.g., Strauß et al. 2010) and even sometimes outside the forest (Glaw and Vences 2007).

The existence of strongly rheophilous tadpoles in species of *Boophis* has been known since the work of Blommers-Schlösser (1979). This author pioneered our understanding of the evolutionary relationships and natural history of Malagasy frogs and described several tadpoles with peculiar morphological characteristics such as an enlarged oral disc, increased number of keratodont rows and papillae, and low tail fin. These larvae were assigned to *Boophis majori*, *Boophis erythrodactylus*, *Boophis mandraka*,and *Boophis* sp. However, in 1979 the true species diversity of *Boophis* was not fully understood (Glaw et al. 2001) and matching of tadpoles to species was difficult without molecular genetic techniques, which resulted in equivocal identity of the tadpoles from these early studies. Tadpoles assigned by Blommers-Schlösser (1979) to *Boophis majori* probably belong to *Boophis marojezensis*, and the identity of tadpoles assigned to *Boophis erythrodactylus* remains uncertain, because this species belongs to a species group which has generalized tadpoles. Subsequent to these early works, Raharivololoniaina et al. (2006) described the tadpoles of *Boophis marojezensis* and *Boophis sibilans* from Andasibe. Glos et al. (2007) described *Boophis schuboeae* tadpoles from Ranomafana and *Boophis ankaratra* tadpoles from Andringitra, and Thomas et al. (2006) described *Boophis andohahela* tadpoles from Ranomafana. More recently, Rasolonjatovo et al. (2010) described the larvae of *Boophis englaenderi*, *Boophis luciae*, and *Boophis vittatus*.

In this study, morphological data on twenty-two strongly rheophilous tadpoles are provided, of which fourteen were previously unknown. Twelve of these larvae belong to candidate species which so far have not been scientifically named.

All these strongly rheophilous tadpoles are characterized by their “streamlined” (i.e., elongated, narrow and flat) body form, their wide oral disc containing many keratodont rows with all posterior rows uninterrupted, their completely keratinized jaw sheaths, of which the lower one is always “ribbed” and the upper one can be absent in some species, and rows of many small rounded marginal papillae with or without a dorsal gap. The absence of many of these characteristics in *Boophis williamsi* tadpoles (Blommers-Schlösser 1979 and Schmidt et al. 2008) is the criteria of excluding them from the present study.

In the context of grouping Malagasy tadpoles into different ecomorphological guilds, some *Boophis* tadpoles have been classified as “suctorial” and “adherent” by Altig and Johnston (1989), and Raharivololoniaina et al. (2006) have classified other *Boophis* tadpoles more in detail according to their morphological characters. However, for a definition of ecomorphological guilds, it is appropriate to include also ecological data. Therefore, we here combine our morphological descriptions with the results of an ecological analysis of the three most abundant strongly rheophilous tadpoles in Ranomafana National Park in the southern central east of Madagascar (*Boophis luciae*, *Boophis marojezensis* [Ca51], and *Boophis andohahela*). Based on habitat characteristics from 30 streams in this rainforest reserve we tested whether the typical morphological characteristics of these tadpoles are indeed associated with a preference for a fast-running stream habitat, as it has been predicted by Blommers-Schlösser (1979).

The use of DNA barcoding to identify amphibian larvae from species-rich tropical communities to the species level has been tremendously successful within the last years (e.g., Thomas et al. 2005). It also has induced a so called “reverse taxonomy” in this vertebrate group (Randrianiaina et al. 2011a), with species new to science discovered first by their larvae rather than their adult stage. The present study confirms this progress by discovering twelve candidate species via tadpole DNA sequences and morphology, many of which are still unknown in their adult stage.

## Materials and methods

### Morphological study of tadpoles

Tadpoles were collected by different kinds of nets having mesh sizes from 2 to 5 mm, depending on the size of the streams, the strength of the water current and the type of substrate. They were euthanised by immersion in chlorobutanol solution, and immediately sorted into homogeneous series based on morphological characters (body shape, relative tail length, eye position and direction, oral disc position, direction and configuration, general color pattern). From each series one specimen was selected and a tissue sample from its tail musculature or fin taken and preserved in 99% ethanol. This specimen is here called “DNA voucher”. All detailed morphological tadpole characterizations and drawings are based on this DNA voucher, whereas variation is sometimes described based on further specimens of the series. After tissue collection, all specimens were preserved in 5% formalin or 70% ethanol. Specimens were deposited in the Zoologische Staatssammlung München, Germany (ZSM). When referring to voucher specimens the original field numbers (FG/MV, FGZC, T, and ZCMV) are usually provided together with the final ZSM catalogue numbers. Tadpoles studied in this paper are summarized in Tables 1 and 2, including data concerning the site and its coordinates, the date of the capture and the collectors.

For detailed morphological examination, especially to determine developmental stages and assess characters of the oral disc, preserved tadpoles were stained slightly with methylene blue. Tadpoles were examined under water, and a few drops of methylene blue were applied to the oral disc, hind limb, spiracle, narial opening and vent tube to better discern their structures. Developmental stages were determined following Gosner (1960).

Morphological descriptions, measurements and drawings were done on digital pictures of the preserved tadpoles taken with a stereomicroscope Zeiss StereoDiscovery V12 connected to a computer, following landmarks, terminology and definitions of Altig and McDiarmid (1999) and Randrianiaina et al. (2011a), except that we predominantly use the term keratodonts instead of labial teeth. The formula of keratodont rows (labial tooth row formula, LTRF) is given according to Altig and McDiarmid (1999). Comparing measurements, we consider them as “almost equal” if ratios of the measured values are 95–96% or 104–105%, “equal” if they are in the range 97–103%, as “almost in the middle” if they are in the range 45–46% or 54–55% and “in the middle” if they are in the range 47–53% (Randrianiaina et al. 2011a). All the measurement data are summarized in Tables 3 to 5 electronic supplement. Most of these data, especially concerning the oral disc, are used for elaborating morphological clusters, into which all tadpoles are classified. The following abbreviations are used: A_1_ (first upper keratodont row), A_2_ (second upper keratodont row), A_2gap_ (medial gap in A_2_), A_3_ (third upper keratodont row), A_4_ (fourth upper keratodont row), A_5_ (fifth upper keratodont row), A_6_ (sixth upper keratodont row), A_7_ (seventh upper keratodont row), A_8_ (eighth upper keratodont row), A_1–8 den_ (density of the keratodonts in row A_1–8_), A_1–8__len_ (length of A_1–8_), A_1–8__num_ (number of keratodonts in A_1–8_), BH (maximal body height), BL (body length), BW (maximal body width), DF (dorsal fin height at midtail), DG (size in rows of the dorsal gap of marginal papillae), DMTH (distance of maximal tail height from the tail-body junction), ED (eye diameter), EH (eyes height – measured from the lower curve of the belly), HAB (height of the point where the axis of the tail myotomes contacts the body – measured from the lower curve of the belly), IND (inter-narial distance), IOD (inter-orbital distance), JW (maximal jaw sheath width), MC (medial convexity of the upper sheath), MCL (length of the medial convexity of the upper sheath), MP (marginal papillae), MTH (maximal tail height), ND (naris diameter), NH (naris height - measured from the lower curve of the belly), NP (naris-pupil distance), OD (oral disc), ODW (maximum oral disc width), P_1_ (first lower keratodont row), P_2_ (second lower keratodont row), P_3_ (third lower keratodont row), P_1–3 den_ (density of the keratodonts in P_1–3_), P_1–3__len_ (length of P_1–3_), P_1–3__num_ (number of keratodonts in P_1–3_), PCA (Principal Component Analysis), RN (rostro-narial distance), SBH (distance between snout and the point of maximal body height), SBW (distance between snout and the point of maximal body width), SE (snout-eye distance), SH (spiracle height – measured from the lower curve of the belly), SL (spiracle length), SMP (submarginal papillae), SS (snout-spiracle distance), SV (spiracle-vent distance), TAL (tail length), TH (tail height at the beginning of the tail), THM (tail height at midtail), Thorn-pap (thorn-shaped papillae), TL (total length), TMH (tail muscle height at the beginning of the tail), TMHM (tail muscle height at midtail), TMW (tail muscle width at the beginning of the tail), LR (number of the lower rows of keratodonts), UR (number of the upper rows of keratodonts), VF (ventral fin height at midtail), VG (size in rows of the ventral gap of marginal papillae), VL (vent tube length).

In tadpoles of many frog species, pigmentless parts of the body wall become detached and apparently separated by a liquid-filled cavity from the underlying pigmented parts of the skin and the inner organs (among Malagasy frogs, for instance extremely expressed in the tadpoles of some *Scaphiophryne*; see Grosjean et al. 2007). These cavities probably represent lymphatic sacs or sinuses but this hypothesis has not been verified in most anuran species. In the rheophilous *Boophis* tadpoles, the extension of this detachment of a transparent part of the body wall appears to be characteristic for some species and candidate species. The difference often refers to the extension and ease to recognize this transparent area of the body wall, and we therefore use terms like recognizable vs. poorly recognizable rather than present vs. absent when referring to this structure, although there are clearly pronounced differences in its expression among some species.

**Table 1.** Summary of localities with geographic coordinates, and collection dates, of tadpole specimens studied herein.

Locality	Site	Species	Coordinates	Date	Collectors
Ankijagna Lalagna		*Boophis sambirano*[Ca49]	14°14.055'S, 48°58.732'E 1187 m a.s.l.	08.06.2010	D.R. Vieites, F.M. Ratsoavina, A.S. Rasamison, A . Rakotoarisoa, M. Vences,R.D. Randrianiaina
Ambohitsara		*Boophis albipunctatus*	21°21.431'S, 47°48.941'E 294 m a.s.l.	03.03.2007	A. Strauß, J. Glos, E. Reeve,T. Rasolonjatovo-H., S. Ndriantsoa, M. Vences,R.D. Randrianiaina
Ambinanitelo		*Boophis marojezensis*[Ca52]	14°13.524'S, 48°57.808'E 1182 m a.s.l.	09.06.2010	D.R. Vieites, F.M. Ratsoavina, A.S. Rasamison, A . Rakotoarisoa, M. Vences,R.D. Randrianiaina
Ambinanitelo		*Boophis sambirano* [Ca50]	14°13.524'S, 48°57.808'E 1182 m a.s.l.	09.06.2010	D.R. Vieites, F.M. Ratsoavina, A.S. Rasamison, A . Rakotoarisoa, M. Vences,R.D. Randrianiaina
An'Ala	Andohanisity	*Boophis mandraka*[Ca46]	18°55.156'S, 48°29.278'E 889 m a.s.l.	08.02.2006	C. Patton, D.R. Vieites,J. Patton, L. Raharivololoniaina, M. Vences, R.D. Randrianiaina
Andasibe Special Reserve	Analamazaotra river	*Boophis sibilans*	18°55.900'S, 48°25.733'E 900 m a.s.l.	04.12.2001	L. Raharivololoniaina, M Vences
Between Antsohihy and Bealanana	Anjingo river	*Boophis sambirano*[Ca47]	14°44.929'S, 48° 29.491'E 925 m a.s.l.	07.06.2010	D.R. Vieites, F.M. Ratsoavina, A.S. Rasamison, A . Rakotoarisoa, M. Vences,R.D. Randrianiaina
Between Antsohihy and Bealanana	Anjingo river	*Boophis sambirano*[Ca48]	14°44.929'S, 48°29.491'E 925 m a.s.l.	07.06.2010	D.R. Vieites, F.M. Ratsoavina, A.S. Rasamison, A . Rakotoarisoa, M. Vences, R.D. Randrianiaina
Manongarivo Special Reserve	Camp Norbert	*Boophis sambirano*	13°56.053'S, 48°27.028’E 288 m a.s.l.	31.01.2003	F. Glaw, M. Vences, R.D. Randrianiaina
Marojejy National Park	Camp Mantella	*Boophis vittatus*	14°26.972'S, 49°47.214'E 327 m a.s.l.	14.02.2005	F. Glaw, M. Vences, R.D. Randrianiaina
Marojejy National Park	Camp Marojejia	*Boophis englaenderi*	14°26.070'S, 49°45.638'E 740 m a.s.l.	18.02.2005	F. Glaw, M. Vences, R.D. Randrianiaina
Marojejy National Park	Camp Mantella	*Boophis englaenderi*[Ca23]	14°26.972'S, 49°47.214'E 327 m a.s.l.	19.02.2005	F. Glaw, M. Vences, R.D. Randrianiaina
Marojejy National Park	Camp Mantella	*Boophis marojezensis*[Ca25]	14°26.972'S, 49°47.214'E 327 m a.s.l.	19.02.2005	F. Glaw, M. Vences, R.D. Randrianiaina
Marojejy National Park	Camp Mantella	*Boophis marojezensis*[Ca26]	14°26.972'S, 49°47.214'E 327 m a.s.l.	19.02.2005	F. Glaw, M. Vences, R.D. Randrianiaina
Marojejy National Park	Camp Mantella	*Boophis sibilans*	14°26.972'S, 49°47.214'E 327 m a.s.l.	19.02.2005	F. Glaw, M. Vences, R.D. Randrianiaina
Ranomafana National Park	Ambatolahyriver	*Boophis andohahela*	21°14.897'S, 47°25.769'E 867 m a.s.l.	27.07.2009	R.D. Randrianiaina
Ranomafana National Park	Ambatolahyriver	*Boophis marojezensis*[Ca51]	21°14.897'S, 47°25.769'E 867 m a.s.l.	27.07.2009	R.D. Randrianiaina
Ranomafana National Park	Ambatolahyriver	*Boophis schuboeae*	21°14.897'S, 47°25.769'E 867 m a.s.l.	27.07.2009	R.D. Randrianiaina
Ranomafana National Park	Imaloka	*Boophis marojezensis*[Ca51]	21°14.529'S, 47°27.938'E 957 m a.s.l.	01.03.2007	A. Strauß, J. Glos, E. Reeve,T. Rasolonjatovo-H., S. Ndriantsoa,M. Vences, R.D. Randrianiaina
Ranomafana National Park	In a poolbelow waterfall	*Boophis schuboeae*		11.02.2003	M. Teschke, M. Vences
Ranomafana National Park	Marihy avaratra	*Boophis luciae*	21°15.806'S, 47°25.548'E 1144 m a.s.l.	20.02.2007	A. Strauß, J. Glos, E. Reeve,T. Rasolonjatovo-H., S. Ndriantsoa,M. Vences, R.D. Randrianiaina
Ranomafana National Park	Marihy avaratra	*Boophis mandraka*[Ca38]	21°15.806'S, 47°25.548'E 1144 m a.s.l.	02.02.2007	A. Strauß, J. Glos, E. Reeve,T. Rasolonjatovo-H., S. Ndriantsoa,M. Vences, R.D. Randrianiaina
Ranomafana National Park	Talatakely	*Boophis luciae*	21°15.846'S, 47°25.161'E 966 m a.s.l.	24.02.2006	L. Raharivololoniaina,A.F. Ranjanaharisoa,T.J. Razafindrabe, D.R. Vieites, J. Patton, C. Patton, M. Vences, R.D. Randrianiaina
Ranomafana National Park	Talatakely	*Boophis marojezensis*[Ca51]	21°15.846'S, 47°25.161'E 966 m a.s.l.	24.02.2006	L. Raharivololoniaina,A.F. Ranjanaharisoa,T.J. Razafindrabe, D.R. Vieites, J. Patton, C. Patton, M. Vences, R.D. Randrianiaina
Ranomafana National Park	Sahateza(Pond Donald)	*Boophis ankaratra*	21°15.476'S, 47°21.583'E 1016 m a.s.l.	03.03.2007	A. Strauß, J. Glos,E. Reeve,T. Rasolonjatovo-H., S. Ndriantsoa,M. Vences, R.D. Randrianiaina
Ranomafana National Park	Vatoharana	*Boophis andohahela*	21°17.338'S, 47°25.765'E 1016 m a.s.l.	24.03.2007	A. Strauß, J. Glos, E. Reeve,T. Rasolonjatovo-H., S. Ndriantsoa,M. Vences, R.D. Randrianiaina
Tsaratanana Strict Nature Reserve	Antevialambazaha	*Boophis marojezensis*[Ca53]	14°10.455'S, 48°56.714'E 1699 m a.s.l.	10.06.2010	D.R. Vieites, F.M. Ratsoavina, A.S. Rasamison, A . Rakotoarisoa, M. Vences, R.D. Randrianiaina

**Table 2.** Collection numbers and Genbank accession numbers of the tadpoles studied. FG/MV, FGZC, LR, T, TAD, ZCMV (field numbers), ZSM (Zoologische Staatssammlung München). Missing accession numbers indicate that sequences were too short or of poor quality and were therefore not submitted to Genbank, or that they will be submitted to Genbank in the course of future studies.

**Species**	**Locality**	**ZSM- and Field number**	**Accession number**
*Boophis englaenderi*	MaroJeJy National Park	FGZC 2244- ZSM 623/2008	HM769921
*Boophis englaenderi* [Ca23]	MaroJeJy National Park	FGZC 2957- ZSM 1632/2007	JQ518193
		FGZC 2241- ZSM 1499/2007	---
		FGZC 2243- ZSM 527/2008	FJ559144
		FGZC 2248- ZSM 1508/2007	---
		FGZC 2250- ZSM 1502/2007	---
		FGZC 2252- ZSM 1503/2007	---
		FGZC 2257- ZSM 529/2008	---
		FGZC 2260- ZSM 530/2008	---
		FGZC 2273- ZSM 1514/2007	---
		FGZC 2275- ZSM 1516/2007﻿	---﻿
*Boophis andohahela*	Ranomafana National Park	T 60- ZSM 912/2007	GU974437
		T 107- ZSM 1319/2007	GU974422
		T 125- ZSM 1321/2007	GU974423
		T 127- ZSM 1162/2007	GU974424
		T 131- ZSM 1351/2007	GU974425
		T 150- ZSM 910/2007	GU974427
		T 222- ZSM 566/2007	GU974435
		T 428- ZSM 998/2007	GU974449
		T 09/273- ZSM 282/2009﻿	---﻿
*Boophis ankaratra*	Ranomafana National Park	FGMV 2003.1698- ZSM 816/2004	﻿---
		ZCMV 3803- ZSM 168/2008	---
		ZCMV 4917- ZSM 876/2007﻿	GU974476﻿
*Boophis schuboeae*	Ranomafana National Park	FGMV 2002.1800- ZSM 978/2004	﻿DQ068394
		Tad 2004-780- ZSM 1339/2004	---
		Tad 2004-797- ZSM 1356-2004	---
		T 09/980- ZSM 743/2008	---
		T 09/968- ZSM 739/2008	---
		T 09/971- ZSM 740/2008	---
		T 09/998- ZSM 749/2008﻿	---﻿
*Boophis albipunctatus*	Ambohitsara	ZCMV 4942- ZSM 78/2008	﻿GU974373
		ZCMV 4946- ZSM 82/2008﻿	GU974374﻿
*Boophis sibilans*	An'Ala	ZCMV 3450- ZSM 1754/2007	---
*Boophis sibilans*	Andasibe	LR 269- ZSM 557/2004	DQ792492
*Boophis sibilans*	MaroJeJy National Park	FGZC 2956- ZSM 1631/2007	JQ518194
*Boophis luciae*	Ranomafana National Park	T 176- ZSM 792/2007	﻿---
		T 177- ZSM 593/2007	GU975090
		T 178- ZSM 541/2007	GU975094
		T 179- ZSM 976/2007	---
		T 224- ZSM 264/2007	---
		T 430- ZSM 274/2007	GU975096
		ZCMV 3619- ZSM 1587/2006	HM769939
		ZCMV 3631- ZSM 1588/2006	HM769940
		ZCMV 3686- ZSM 634/2008	HM769938
		ZCMV 4024- ZSM 688/2007	---
		ZCMV 5146- ZSM 730/2007﻿	---﻿
*Boophis sambirano*	Manongarivo Special Reserve	FGMV 2002.1904- ZSM 678/2004	﻿EU717863
		FGMV 2002.1902- ZSM 672/2004﻿	EU717861﻿
*Boophis mandraka* [Ca38]	Ranomafana National Park	ZCMV 4261- ZSM 456/2007	FJ559153
*Boophis mandraka* [Ca46]	An'Ala	ZCMV 3479- ZSM 1784/2007	JQ518195
*Boophis sambirano* [Ca47]	Between Antsohihy and Bealanana	ZCMV 13105- ZSM 482/2010	﻿JQ518203
		ZCMV 13110- ZSM 486/2010﻿	JQ518204﻿
*Boophis sambirano* [Ca48]	Between Antsohihy and Bealanana	ZCMV 13107- ZSM 484/2010	﻿JQ518206
		ZCMV 13108- ZSM 485/2010	JQ518207
		ZCMV 13109- ZSM 485/2010﻿	JQ518205﻿
*Boophis sambirano* [Ca49]	AnkiJagna Lalagna	ZCMV 13150- ZSM 523/2010	﻿JQ518209
		ZCMV 13155- ZSM 528/2010	JQ518208
		ZCMV 13156- ZSM 529/2010﻿	JQ518210﻿
*Boophis sambirano* [Ca50]	Ambinanitelo	ZCMV 13171- ZSM 544/2010	﻿JQ518212
		ZCMV 13172- ZSM 545/2010	JQ518211
		ZCMV 13173- ZSM 546/2010	JQ518213
		ZCMV 13174- ZSM 547/2010﻿	JQ518214﻿
*Boophis marojezensis*	MaroJeJy National Park	FGZC 2277- ZSM 1528/2007	﻿JQ518196
		FGZC 2953- ZSM 1628/2007﻿	JQ518199﻿
*Boophis marojezensis* [Ca25]	MaroJeJy National Park	FGZC 2929- ZSM 1611/2007	FJ559146
*Boophis marojezensis* [Ca26]	MaroJeJy National Park	FGZC 2930- ZSM 1612/2007	JQ518197
*Boophis marojezensis* [Ca51]	Ranomafana National Park	T 394- ZSM 1008/2007	﻿GU974657
		T 432- ZSM 117/2007	GU974658
		T 09/1088- ZSM 779/2008	---
		T 09/1091- ZSM 780/2008	---
		T 09/1094- ZSM 781/2008	---
		ZCMV 3691- ZSM 267/2008	---
		ZCMV 3629- ZSM 318/2008	---
		ZCMV 3635- ZSM 232/2008	---
		ZCMV 3690- ZSM 266/2008	---
		ZCMV 3742- ZSM 481/2008	---
		ZCMV 4203- ZSM 401/2007	---
		ZCMV 4264- ZSM 457/2007	GU974654
		ZCMV 4376- ZSM 1453/2007	GU974647
		ZCMV 4531- ZSM 532/2007	GU974648
		ZCMV 4541- ZSM 504/2007	GU974650
		ZCMV 4547- ZSM 1390/2007	GU974651
		ZCMV 4550- ZSM 509/2007	GU974652
		ZCMV 4931- ZSM 838/2007	GU974656
		ZCMV 5098- ZSM 913/2007	GU974646
		ZCMV 5986- ZSM 1212/2007	GU974655
		ZCMV 1395- ZSM 0025/2007	GU974653
		T 09/1085- ZSM 778/2008﻿	---﻿
*Boophis marojezensis* [Ca52]	Ambinanitelo	ZCMV 13168- ZSM 541/2010	﻿JQ518215
		ZCMV 13169- ZSM 542/2010﻿	---﻿
*Boophis marojezensis* [Ca53]	Tsaratanana Strict Nature Reserve	ZCMV 13200- ZSM 573/2010	﻿JQ518216
		ZCMV 13201- ZSM 574/2010	---
		ZCMV 13202- ZSM 575/2010	---
		ZCMV 13203- ZSM 576/2010	---
		ZCMV 13204- ZSM 577/2010	---
		ZCMV 13205- ZSM 578/2010﻿	JQ518217﻿
*Boophis vittatus*	MaroJeJy National Park	FGZC 2237- ZSM 5219/2005	﻿---
		FGZC 2238- ZSM 1906/2007	JQ518200
		FGZC 2251- ZSM 1907/2007	JQ518201
		FGZC 2914- ZSM 1601/2007﻿	JQ518202﻿﻿

### DNA-based species identification

DNA barcoding was based on a fragment of the mitochondrial 16S rRNA gene, which is known to be sufficiently variable among species of Malagasy frogs (Vences et al. 2005). We amplified a fragment of ca. 550 bp using primers 16Sar-L and 16Sbr-H from Palumbi et al.(1991), or a shorter fragment of ca. 400 bp using the newly developed specific mantellid primers 16S-Frog-L1 (CAT AAT CAC TTG TTC TTT AAA) and 16S-Frog-H1 (GAT CCA ACA TCG AGG TCG). PCR was carried out with standard protocols (Vences et al. 2005) and sequences resolved on automated sequencers. Sequences were preliminarily identified using BLAST searches against a near-complete database of sequences of adult Malagasy frog species. Results were subsequently verified by manually aligning and comparing sequences to the closest hits in the data base. Identification was considered to be unequivocal when the tadpole sequence was 99–100% identical to an adult specimen from the same geographical region, and clearly less similar to all sequences from other species. When no identity with adult specimens was found and divergence was >3% we considered the corresponding tadpoles to belong to undescribed candidate species. Newly determined DNA sequences were deposited in Genbank (accession numbers JQ518193- JQ518217).

Candidate species nomenclature followed the scheme developed by Padial et al. (2010). We use the binomial species name of the closely related species, followed in square by the abbreviation “Ca” with an attached numerical code referring to the particular candidate species, and at first mention terminating with the author name and the year of publication of the article in which the lineage was first discovered for few species, or the Genbank accession number of a DNA sequence of a reference specimen for others. Further in the text, we abbreviate the candidate species name just by using the binomial species name followed in square brackets by the abbreviation “Ca” and its numerical code.

### Ecological study of tadpoles

During a study on stream tadpole communities in Ranomafana National Park (RNP) in the south eastern escarpment of Madagascar, we exhaustively sampled 33 stream sections for tadpoles (Strauß et al. 2010). Each section spanned 30 m and the sampling process was conducted separately for all available microhabitats within the section. We aimed to exhaustively sample tadpoles using dip nets of different sizes and materials, adjusted to obtain optimal sampling results for each stream. Sampling started downstream, and depending on stream width two to five people processed slowly on the same level upstream while dip-netting as much as reasonably possible all tadpoles in all microhabitats. These microhabitats were predefined subject to underground substrate (rock, gravel, leaves, sand) as well as separately by the stream velocity categories “fast” (obviously running) and “slow” (almost stagnant). Habitat variables were recorded at two spatial levels: *(1)* habitat variables of possible importance for breeding site (stream) choice of frog species and *(2)* proportion of microhabitats available within the streams.

We used data from this study for an exemplary analysis of breeding site choice and microhabitat use of syntopic species of strongly rheophilous tadpoles. To identify the habitat variables of the stream and the surrounding forest that may be important for breeding site choice, we performed a principal component analysis (PCA) and plotted species according to their incidence as supplementary variables in the PCA biplot. For PCA, we used all ten habitat variables of all 33 streams sampled during the tadpole community study. PCA was run on the correlation matrix in order to standardize for the influence of unequal variance. To evaluate data outliers and linear interdependence of variables, box-plots and pair-plots (Zuur et al. 2007) were used. As PCA requires multinormality of data, box-cox-power-transformations (Box and Cox 1964) were applied when necessary. The number of meaningful PCs was estimated by a scree plot (Zuur et al. 2007). PCA and correlation with species incidence was evaluated using the dimdesc function in package FactoMineR (Lê et al. 2008).

To analyze the use of microhabitats within streams, we first constructed graphs of raw data to display the species specific distribution between microhabitats. In order to quantify true preferences for microhabitats, Ivlev's electivity index (E, Ivlev 1961) was calculated for each strongly rheophilous *Boophis* species occurring in RNP. E is defined as E=(r-p)/(r+p) with r being the proportions of the microhabitats used and p the proportion of microhabitats available. To test whether the E values differ for the single species, a factorial ANOVA was run with E as dependent variable and the factors “microhabitat” and “species” as independent variables. This provides information whether E is different for the different microhabitats, whether E differs between species, and, if interactions could be included in model, whether the effect of the one factor depends on the level of the other factor. To avoid possible overparameterisation caused by large numbers of interactions (Crawley 2007), we removed the interaction term from the model and performed ANOVAs of subsets of the data to closer evaluate differences in preferences between species within specific microhabitats (interactions). Only the three abundant species were included in this analysis. Also, for each species only streams with at least eight specimens of the respective species were included in the analysis to reduce the influence of many high avoidance values due to a general low number of tadpoles in a stream.

Statistical analysis were performed in R 2.9.2 (R Development Core Team 2009) including libraries car (Fox et al. 2008) and FactoMineR (Lê et al. 2008).

## Results

### Tadpole descriptions

We here provide a summary of the most important morphological characteristics of one representative species per species group, and brief accounts for all other species and candidate species in which we mainly emphasize their difference to the species described more completely, or to other species belonging to the same group. Standardized, detailed descriptions and assessments of variation for all species and candidate species are found in the electronic supplement. Original measurements and ratios are given in Tables 3–5 which are equally included as electronic supplement.

**Figure 1.**
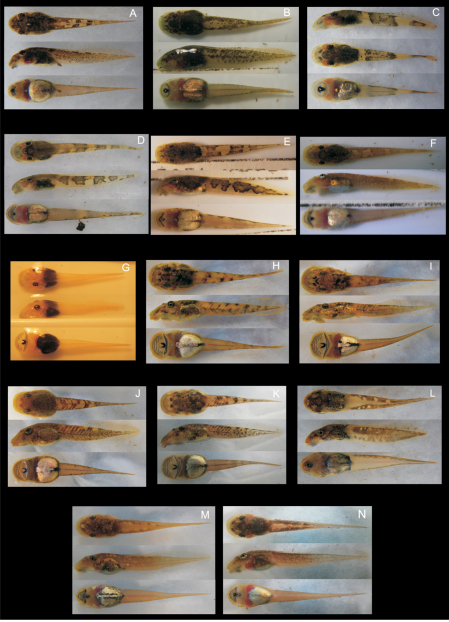
Coloration in life of strongly rheophilous tadpoles of *Boophis* (dorsal, lateral and ventral views): **A** *Boophis andohahela* (T 09/273-ZSM 282/2009) **B** *Boophis ankaratra* (ZCMV 4917-ZSM 876/2007) **C** *Boophis schuboeae* (T 09/980-743/2008) **D** *Boophis sibilans* (ZCMV 11548 - to be catalogued in ZSM) **E** *Boophis luciae* (ZCMV 11548-to be catalogued in ZSM) **F** *Boophis albipunctatus* (ZCMV 4946-ZSM 82/2008) **G** *Boophis mandraka* [Ca38](ZCMV 4261-ZSM 456/2007) **H** *Boophis sambirano* [Ca47](ZCMV 13105-ZSM 482/2010) **I** *Boophis sambirano* [Ca48](ZCMV 13109-ZSM 486/2010) **J** *Boophis sambirano* [Ca49](ZCMV 13155-ZSM 528/2010) **K** *Boophis sambirano* [Ca50](ZCMV 13172-ZSM 545/2010) **L** *Boophis marojezensis* [Ca51] (ZCMV 13550-ZSM 721/2010) **M** *Boophis marojezensis* [Ca52] (ZCMV 13168-ZSM 541/2010) **N** *Boophis marojezensis* [Ca53] (ZCMV 13200-ZSM 573/2010).

**Figure 2.**
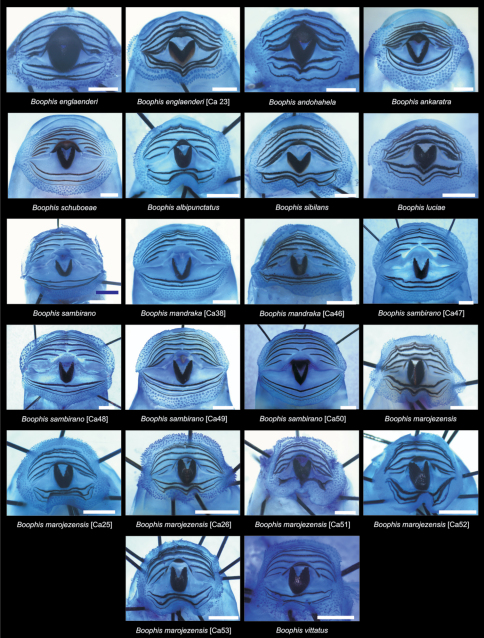
Pictures of the oral discs of the voucher specimens (stained with methylene blue for better visibility of morphological structures). Scale bars represent 1 mm.

### *Boophis luteus* group

This group is characterized by tadpoles having a generalized oral disc without lateral emargination and ventral gap of papillae, but the dorsal gap is wide to very wide. The anterior margin of the oral disc is a continuation of the snout. Usually A_1_ is uninterrupted and P_1_ is interrupted, except the three species described herein which are the ones in the group having the strongest expression of adaptations that we interpret as rheophilous. The jaw sheaths are very strong with smooth surface and completely or partially keratinized in some species. The upper sheath is always made up by a medial convexity. Dorsolateral glands which exist in some other *Boophis* tadpoles are absent.

***Boophis englaenderi* Glaw & Vences, 1994**

**Figure 3.**
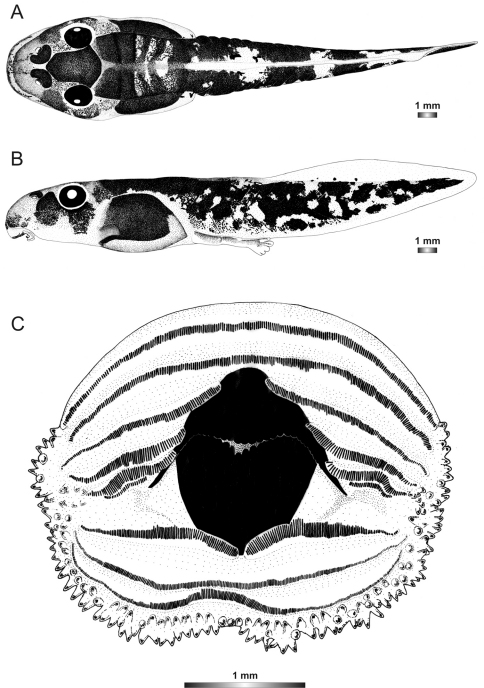
Drawings of the preserved DNA voucher tadpole of *Boophis englaenderi* (FGZC 2244-ZSM 623/2008): **A** Dorsal view **B** Lateral view **C** Oral disc.

Morphological data were assessed in one tadpole (Figures 2 and 3) in developmental stage 36 (field number FGZC 2244; ZSM 623/2008, BL 11.8 mm, TL 25.4 mm, accession number HM769921) from Marojejy National Park (previously described by Rasolonjatovo Hiobiarilanto et al. 2010). The 16S rDNA sequence of this specimen is 99.5% identical to a reference sequence of an adult *Boophis englaenderi* (accession FJ559124) from Marojejy.

The tadpoles of this species have an elliptical body, a flatly rounded snout in dorsal view and a short tail. The distance between eyes is wide and nares are very large, round, positioned very high dorsally, and situated nearer to snout than to eye and at eye level. LTRF is 6(3–6)/3(1). The upper jaw sheath is totally keratinized with rounded serrations, moderately wide with a very short widely rounded medial convexity. The lower sheath is V-shaped, completely keratinized and partially hidden by the upper one. Both jaw sheaths have a smooth surface.

In preservative, the tadpole is generally dark brown. Dark brown spots condensed to form a hexagonal mark above the neurocranium; a dark semicircular patch situated posterior to each narial opening and dark patches between the vertebral area and the abdominal region are present. The snout is spotted. The transversal lines between the vertebral area and the abdominal region are perceivable which make the domino-like structure on this noticeable. The dorsal part of the tail muscle has five dark brown and four light alternating bands. The prominent dark brown band is the extension of the patches between the vertebral area and the abdominal region. The myosepta are visible on the dorsal part of the tail. Laterally, the jugal area is covered by dense dark brown patches and the dorsolateral part of the flank is identical to the dorsal pattern; the ventrolateral part is pale and the abdominal region is very dark leaving an opaque discernible spiracle. Ventrally, oral disc, gular and branchial regions are pale; the venter is more or less transparent and the intestinal coils are perceptible with a regularly spiral shape. The tail musculature is pale and covered by dark brown spots which condense to form reticulations. Fins are transparent, with few brown spots on the dorsal fin, and the ventral fin is free from pigment.

***Boophis englaenderi* [Ca23 Vieites et al. 2009*]***

**Figure 4.**
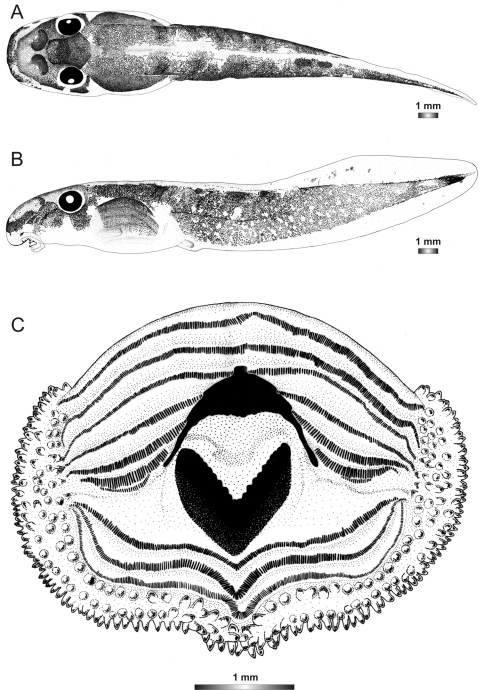
Drawings of the preserved DNA voucher tadpole of *Boophis englaenderi* [Ca23](FGZC 2957-ZSM 1632/2007): **A** Dorsal view **B** Lateral view **C** Oral disc.

Morphological data were assessed in one tadpole (Figures 2 and 4) in developmental stage 30 (field number FGZC 2957, ZSM 1632/2007, BL 10.5 mm, TL 29.5 mm, Genbank accession number JQ518193) from Marojejy National Park. The 16S rDNA sequence of this specimen is 94% identical to a reference sequence of an adult *Boophis englaenderi* (accession AY848474) from Ilampy. Nine other voucher specimens agree in morphology with the voucher specimen described herein.

The external morphology of this tadpole has a very close similarity with that of *Boophis englaenderi*, except that it has a distinctly longer tail (TAL/BL 183% *vs*. 153%) and a lighter pigmentation. Additional differences between the two tadpoles are found in the oral disc structure. It is bulged laterally and has one more interrupted upper keratodont row and a first uninterrupted lower row giving the keratodont row formula LTRF 7(3–7)/3 *vs*. 6(3–6)/3(1). The number of papillae is higher than in*Boophis englaenderi* with 175 marginal papillae (*vs*. 128), and 94 submaginal papillae (*vs*. 33), although the examined tadpole is still in a developmental stage inferior to that of the examined tadpole of *Boophis englaenderi*. The submarginal papillae are complete on the lower labium. This tadpole is also characterized by a light brown coloration in preservative. The jugal area is covered by scarce light brown patches, and the tail musculature is covered by light brown spots which group in some areas to form patches or sparse reticulations. The intestinal coils are visible. The examination of nine other voucher specimens (see Table 2) confirms the differences to *Boophis englaenderi*.

***Boophisandohahela* Andreone, Nincheri & Piazza, 1995**

**Figure 5.**
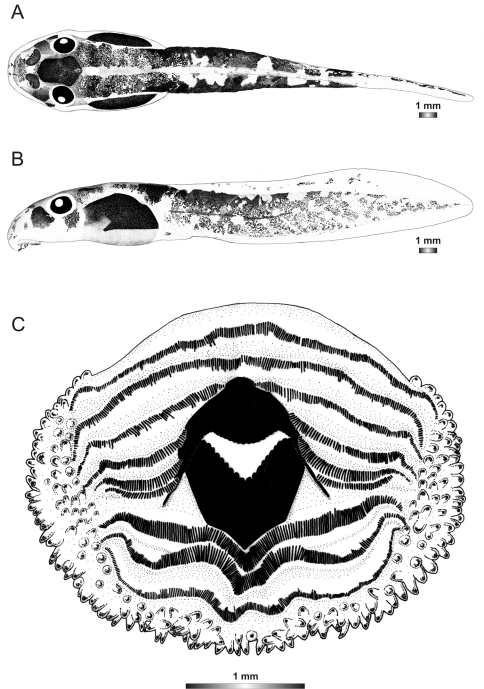
Drawings of the preserved DNA voucher tadpole of *Boophis andohahela* (T 428-ZSM 998/2007): **A** Dorsal view **B** Lateral view **C** Oral disc.

Morphological data were assessed in one tadpole (Figures 2 and 5) in developmental stage 26 (field number T 428; ZSM 998/2007, BL 11.8 mm, TL 25.4 mm, Genbank accession number GU974449) from Ranomafana National Park. The 16S rDNA sequence of this specimen is 100% identical to a reference sequence of an adult *Boophis andohahela* (accession AY848456) from the same locality. Five out of six other voucher specimens have the morphological characteristics of this species, whereas one tadpole has a difference in the oral disc configuration.

The general morphology of this tadpole is similar to that of *Boophis englaenderi* and *Boophis englaenderi* [Ca23], but it is characterized by the presence of a white patch posterior to the hexagonal mark above the neurocranium in life and even in preservative (Figure 1). The non-visibility of its intestinal coils is shared with *Boophis englaenderi*. The LTRF 6(3–6)/3 is identical to that of some specimens of *Boophis englenderi* but differs from that of *Boophis englaenderi* [Ca 23]. On the other hand, the absence of papillae on the ventral area of the lower labium is similar to that of *Boophis englaenderi*. The oral disc of this tadpole has a slightly developed lateral bulge.

### *Boophis albipunctatus* group

This group is characterized by tadpoles having an enlarged oral disc without lateral emargination (but bulged laterally in some species) and ventral gap of marginal papillae. The dorsal gap is moderately wide. The anterior margin of the oral disc is separated by a deep crevice to the snout; i.e., the entire margin is free from the snout. LTRF 8(5–8)/3 or 7(5–7)/3. The jaw sheaths are moderately strong and completely keratinized. The upper sheath has a medial convexity in some species. The lower sheath is U or V-shaped and ribbed. Dorsolateral glands are present.

***Boophis ankaratra* Andreone, 1993**

**Figure 6.**
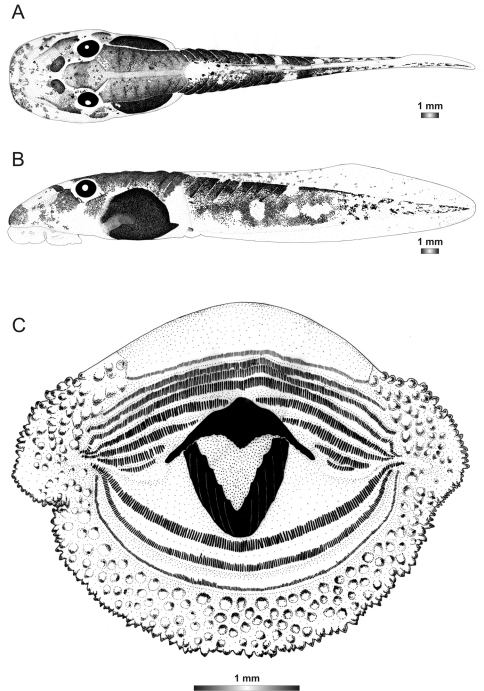
Drawings of the preserved DNA voucher tadpole of *Boophis ankaratra* (ZCMV 4917-ZSM 876/2007): **A** Dorsal view **B** Lateral view **C** Oral disc.

Morphological data were assessed in one tadpole (Figures 2 and 6) in developmental stage 28 (field number ZCMV 4917, ZSM 876/2007, BL 11.3 mm, TL 25.5 mm, Genbank accession number GU974476) from Ranomafana National Park. The 16S rDNA sequence of this specimen is 100% identical to a reference sequence of an adult *Boophis ankaratra* (accession AJ315909) from Mandraka. Two other voucher specimens possess the typical morphological characters of the species.

This tadpole can be differentiated from *Boophis luteus* goup tadpoles by the general state of the oral disc. It is characterized by an enlarged and laterally bulged oral disc. There is a double row of marginal papillae interrupted by a moderately wide dorsal gap. Papillae are short, small, conical with protuberance, and their tip is rounded. There are 148 and 190 marginal and submarginal papillae, respectively. The LTRF is 8(5–8)/3 and A_1_ is moderately long. The jaw sheaths are moderately strong and totally keratinized. The upper sheath is characterized by a short narrowly pointed medial convexity. The lower sheath is U-shaped, ribbed, higher than wide, and partially hidden by the upper one.

In life this tadpole is generally dark brown. Dorsally, body and tail covered by dense brown spots. A hexagonal mark above the neurocranium and a dark semicircular patch posterior to each narial opening are obvious. The domino-like structures between the vertebral area and the abdominal region are recognizable. Few irregular dark blotches and silvery spots scattered on the skin. Laterally, jugal area is covered by dense brown patches and the abdominal region is very dark leaving a transparent noticeable spiracle. The tail musculature is yellowish and covered by sparse brown spots which coalesce to form patches. Their density diminishes toward the tail tip. Fins are transparent with few brown blotches on the dorsal fin and the ventral fin is almost free from pigment. Ventrally, intestinal coils are invisible (Figure 1). In preservative, the tadpole is similar except that it is paler and the silver tissue which covers the heart and the venter has become whitish.

***Boophis schuboeae* Glaw & Vences, 2002**

**Figure 7.**
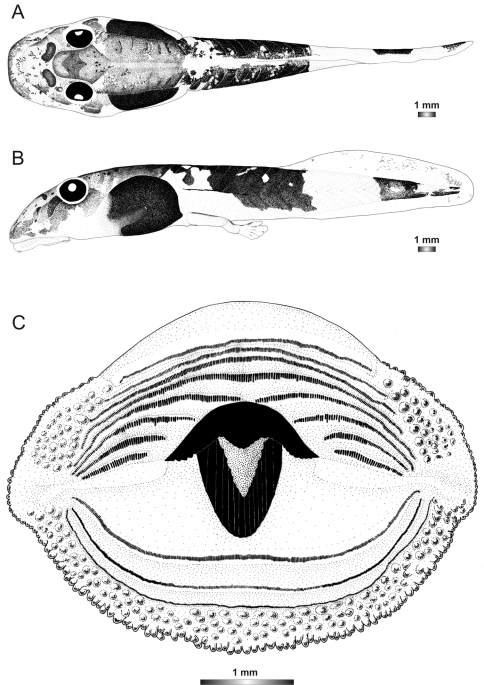
Drawings of the preserved DNA voucher tadpole of *Boophis schuboeae* (FG/MV 2003.1800-ZSM 978/2004): **A** Dorsal view **B** Lateral view **C** Oral disc.

Morphological data were assessed in one tadpole (Figures 2 and 7) in developmental stage 36 (field number FGMV 2002–1800, ZSM 978/2004, BL 12.1 mm, TL 25.5 mm, Genbank accession number DQ068394) from Ranomafana National Park. The 16S rDNA sequence of this specimen is 100% identical to a reference sequence of an adult *Boophis schuboeae* (accession AJ315912) from the same locality. Six other voucher specimens from the same locality show the typical coloration pattern and oral disc configuration of the species.

The oral disc of the tadpoles belonging to this species is identical to those of *Boophis ankaratra*, except that it has a lower number of rather smaller papillae, and the lateral area where the oral disc folds is free from submarginal papillae. However, the tadpoles of these two species are easy to distinguish by their particular coloration pattern (Figure 1) (see also Glos et al. 2007). *Boophis schuboeae* tadpoles are characterized by the presence of up to four light and three alternating dark bands on the tail musculature. In life, the posterior part of the tail is sometimes with a contrasting orange coloration. Typically the dorsal and ventral fins originate on the tail musculature for *Boophis schuboeae* while they commonly originate on the body-tail junction for *Boophis ankaratra*.

***Boophis albipunctatus* Glaw & Thiesmeier, 1993**

**Figure 8.**
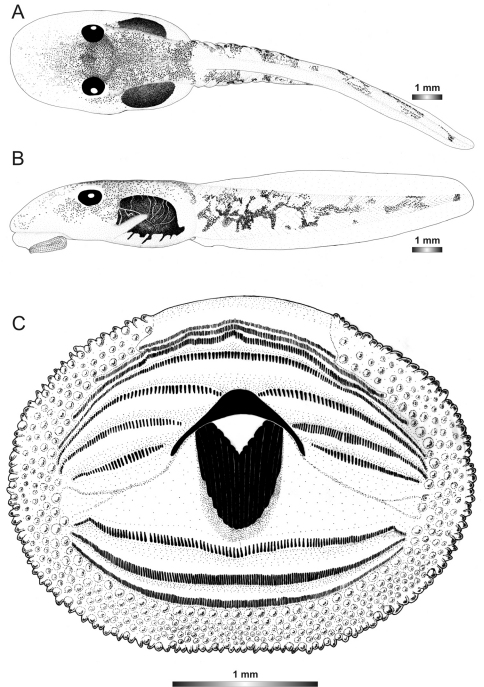
Drawings of the preserved DNA voucher tadpole of *Boophis albipunctatus* (ZCMV 4946-ZSM 82/2008): **A** Dorsal view **B** Lateral view **C** Oral disc.

Morphological data were assessed in one tadpole (Figures 2 and 8) in developmental stage 25 (field number ZCMV 4946, ZSM 82/2008, BL 7.5 mm, TL 15.5 mm, Genbank accession number GU974374) from Ambohitsara-Tsitolaka. The 16S rDNA sequence of this specimen is 99% identical to a reference sequence of a *Boophis albipunctatus* adult specimen (accession AY848446) from Manantantely. One other voucher tadpole of *Boophis albipunctatus* from the same locality is morphologically very similar to the described voucher specimen.

*Boophis albipunctatus* tadpoles can be distinguished from those of *Boophis ankaratra* and *Boophis schuboeae* by the absence of the lateral bulge on the oral disc, the absence of the medial convexity on the upper sheath, the high number of papillae, and the LTRF 7(5–7)/3, but they share the ribbed pattern, the U-shape, and the partially hidden state of the lower jaw sheath. These tadpole are also characterized by their less pigmented state in preservative which makes them easy to identify. The absence of silver pigment covering the heart in life is also typical for these tadpoles.

***Boophis sibilans* Glaw & Thiesmeier, 1993**

**Figure 9.**
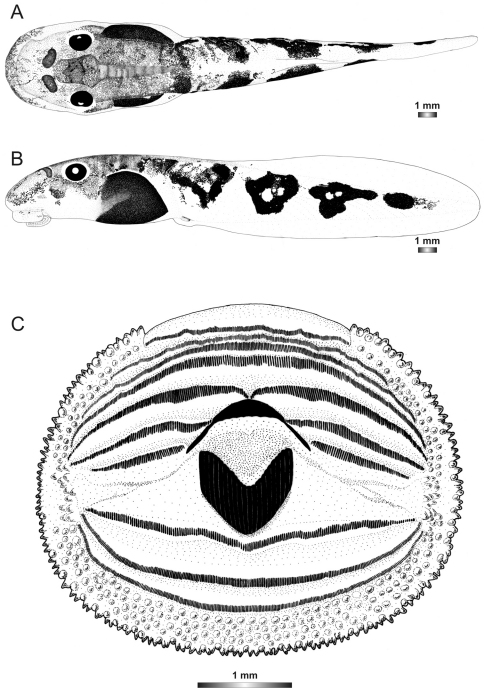
Drawings of the preserved DNA voucher tadpole of *Boophis sibilans* (FGZC 2956-ZSM 1631/2007): **A** Dorsal view **B** Lateral view **C** Oral disc.

Morphological data were assessed in one tadpole (Figures 2 and 9) in developmental stage 29 (field number FGZC 2956, ZSM 1631/2007, BL 11 mm, TL 26 mm, Genbank accession number JQ518194) from Marojejy National Park. The 16S rDNA sequence of this specimen is 99.4% identical to a reference sequence of a *Boophis sibilans* adult specimen (accession AY341718) from Andasibe. Two other voucher tadpoles have similar morphological characteristics.

*Boophis sibilans* tadpoles have the same oral disc feature (absence of lateral bulge, LTRF) as *Boophis albipunctatus*, except for a lower number of submarginal papillae and a V-shaped lower sheath. These tadpoles are characterized by their rather long tail (up to 200% of BL) and their unique tail pattern which is composed of dark spots separated by a clear unpigmented area. The inner part of the spots is usually free from pigment (Figure 1).

***Boophis luciae* Glaw, Köhler, de la Riva, Vieites & Vences, 201*0***

**Figure 10.**
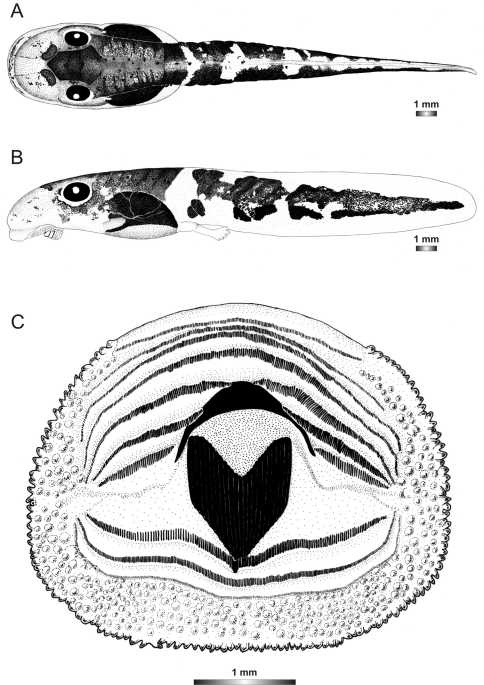
Drawings of the preserved DNA voucher tadpole of *Boophis luciae* (ZCMV 5146-ZSM 730/2007): **A** Dorsal view **B** Lateral view **C** Oral disc.

Morphological data were assessed in one tadpole (Figures 2 and 10) in developmental stage 36 (field number ZCMV 5146, ZSM 730/2007, BL 10.4 mm, TL 22.2 mm, Genbank accession number GU975069) from Ranomafana National Park. The 16S rDNA sequence of this specimen is 100% identical to a reference sequence of a *Boophis luciae* adult specimen (accession AY848444) from the same locality. Ten other voucher tadpoles are morphologically very similar to the described voucher specimen.

The tadpoles of *Boophis luciae* are similar to those of *Boophis sibilans* by their oral disc structure and the general external pattern except that they have a rather short tail. They can be characterized by the state of the spots on the tail musculature which are connected to each other (Figure 1).

### *Boophis mandraka* group

This group is characterized by tadpoles having an enlarged oral disc without lateral emargination and ventral gap of papillae. The dorsal gap of papillae is narrow to very narrow, and the lateral area where the oral disc folds is free of submarginal papillae. The anterior margin of the oral disc is separated by a deep crevice to the snout; i.e., the entire margin is free from the snout. The upper labium has always five uninterrupted and three interrupted keratodont rows, and the three lower rows are always uninterrupted giving a unique LTRF 8(6–8)/3. The upper sheath is always absent. The lower sheaths are moderately strong and completely keratinized, U-shaped, ribed, and higher than wide. Dorsolateral glands are present.

***Boophis sambirano* Vences & Glaw, 2005**

**Figure 11.**
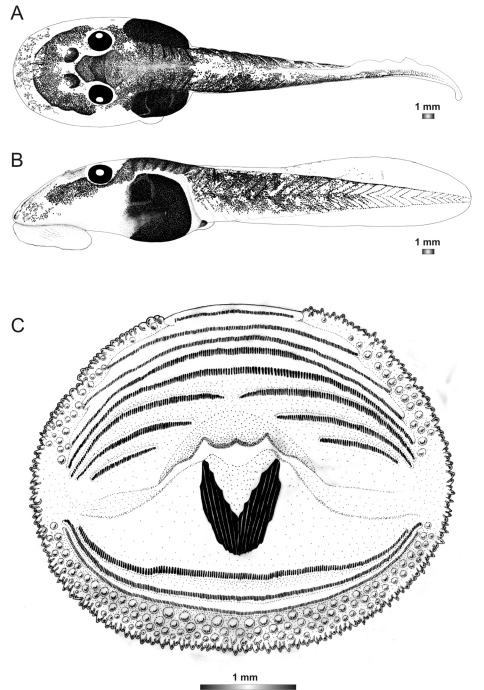
Drawings of the preserved DNA voucher tadpole of *Boophis sambirano* (FG/MV 2002.1904-ZSM 678/2004): **A** Dorsal view **B** Lateral view **C** Oral disc.

Morphological data were assessed in one tadpole (Figures 2 and 11) in developmental stage 25 (field number FG/MV 2002.1902, ZSM 672/2004, BL 6.5 mm, TL 12.7 mm, Genbank accession number EU717861) from a site locally named “Camp Norbert” in Manongarivo Special Reserve. The 16S rDNA sequence of this specimen is 96% identical to a reference sequence of *Boophis sambirano* adult specimen (accession AY848544), and because of this 4% difference its identity and belonging to the “true” *Boophis sambirano* needs further confirmation. Since this specimen was collected next to the type locality of *Boophis sambirano* in Manongarivo, following a parsimonious approach we here assign it to this species, although the large numbers of distinct lineages in *Boophis sambirano* make it likely that yet another candidate species of this complex occurs in Manongarivo. Many non-voucher specimens of the same series present morphological similarities to the voucher specimen.

*Boophis sambirano* tadpoles are easy to distinguish from all other tadpoles described above by the state of their oral disc which has no upper jaw sheath, a short keratodont row A_1_, and a narrow dorsal gap of papillae. The absence of submarginal papillae on the lateral area where the oral disc folds is shared with *Boophis schuboeae*. The tadpoles of this species are also characterized by the extension of an obvious lateral transparent area of the body wall only on the anterior 2/3 of the body, but not surrounding the whole body like in other tadpoles.

***Boophis mandraka* [Ca38 Vieites et al. 2009]**

**Figure 12.**
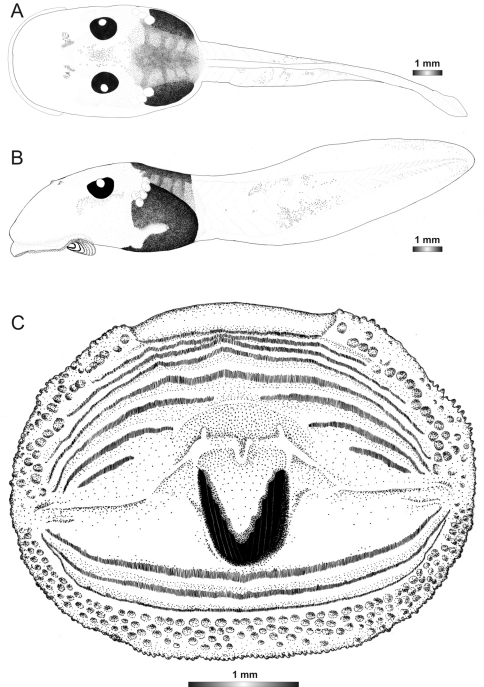
Drawings of the preserved DNA voucher tadpole of *Boophis mandraka* [Ca38](ZCMV 4261-ZSM 456/2007): **A** Dorsal view **B** Lateral view **C** Oral disc.

Morphological data were assessed in one tadpole (Figures 2 and 12) in developmental stage 26 (field number ZCMV 4261, ZSM 456/2007, BL 7.6 mm, TL 15.8 mm, Genbank accession number FJ559153) from Ranomafana National Park. The 16S rDNA sequence of this specimen is 93.3 % identical to a reference sequence of a *Boophis sambirano* adult specimen (accession EU717863) from Manongarivo Special Reserve.

The single tadpole of this candidate species has a similar oral disc structure to *Boophis sambirano* except that it has a slightly wider dorsal gap of papillae (DG/ODW 39% *vs*. 34%). The typical coloration, yellowish in life (Figure 1) and whitish in preservative and the good visibility of the 10 (5 right and 5 left) dorsolateral glands allow its distinction from other tadpoles.

***Boophis mandraka* [Ca46 JQ518195]**

**Figure 13.**
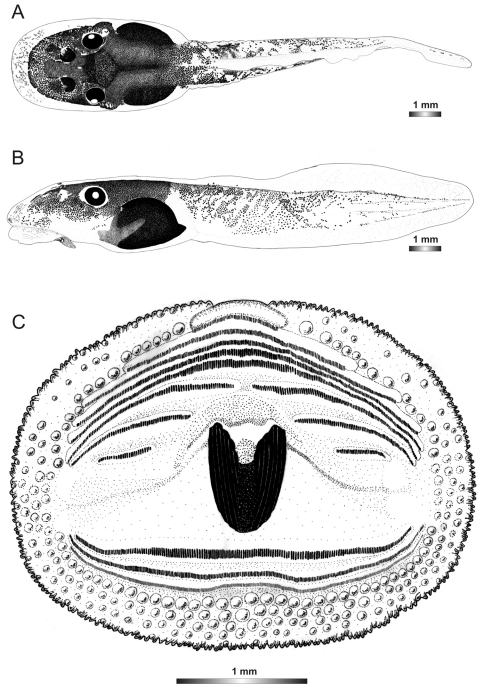
Drawings of the preserved DNA voucher tadpole of *Boophis mandraka* [Ca46](ZCMV 3479-ZSM 1784/2007): **A** Dorsal view **B** Lateral view **C** Oral disc.

Morphological data were assessed in one tadpole (Figures 2 and 13) in developmental stage 25 (field number ZCMV 3479, ZSM 1784/2007, BL 6.8 mm, TL 14.3 mm, Genbank accession number JQ518195) from An'Ala. The 16S rDNA sequence of this specimen is 90.4 % identical to a reference sequence of *Boophis* sp. aff. *mandraka* adult specimen (accession AY848542) from Ilampy.

The oral disc of the single tadpole of this candidate species is similar to those of *Boophis sambirano* and *Boophis mandraka* [Ca38] except that it has the narrowest dorsal gap of papillae with DG 14% of ODW and the shortest A_1_ with 21% of ODW. Within the *Boophis mandraka* group tadpoles, it has also the lowest number of papillae. The external morphology of the single tadpole of this candidate species is similar to that of tadpoles of *Boophis sambirano*, except that the ratio RN/NP is much higher (194 *vs*. 125) and the pigmentation pattern is slightly different.

***Boophis sambirano* [Ca47 JQ518203]**

**Figure 14.**
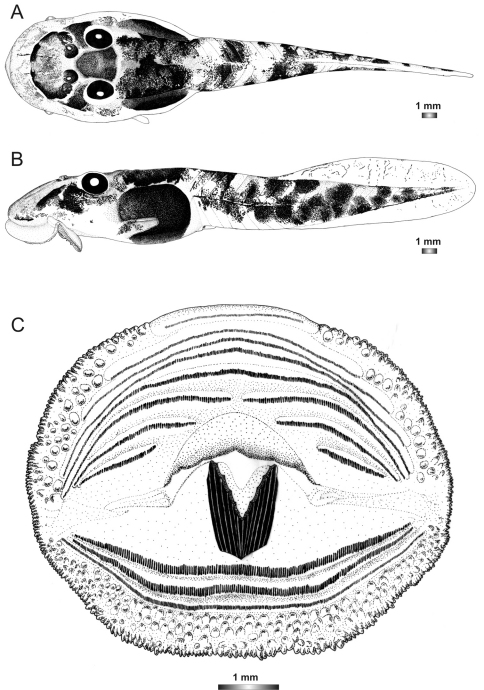
Drawings of the preserved DNA voucher tadpole of *Boophis sambirano* [Ca47](ZCMV 13105-ZSM 482/2010): **A** Dorsal view **B** Lateral view **C** Oral disc.

Morphological data were assessed in one tadpole (Figures 2 and 14) in developmental stage 27 (field number ZCMV 13105, ZSM 482/2010, BL 13.5 mm, TL 27.1 mm, Genbank accession number JQ518203) from Anjingo river (bridge 57 km from Antsohihy to Bealanana). The 16S rDNA sequence of this specimen is 97% identical to a reference sequence of *Boophis sambirano* tadpoles (accession EU717861) from Manongarivo Special Reserve. Two other voucher tadpoles are morphologically very similar to the described voucher specimen.

The tadpoles assigned to this candidate species have a similar oral disc structure as *Boophis sambirano* exept that they have a higher number of marginal papillae (377 *vs*. 248) and of keratodonts on A_3_ (1193 *vs*. 740). These tadpoles have a rather large size in comparison to others of the *Boophis mandraka* group, and their pigmentation pattern distinguishes them also. Their tail musculature is covered by dissipated distinct patches following mainly the lateral tail vein and the myosepta on the anterior half of the tail musculature, and irregularly dispersed on the posterior half (Figure 1), whereas it is just covered by dense spots on the anterior half in *Boophis sambirano* tadpoles. The dorsal fin of these tadpoles begins usually on the anterior 1/5 of the tail musculature, *vs*. beginning more or less at the dorsal body-tail junction in *Boophis sambirano*.

***Boophis sambirano* [Ca48 JQ518205]**

**Figure 15.**
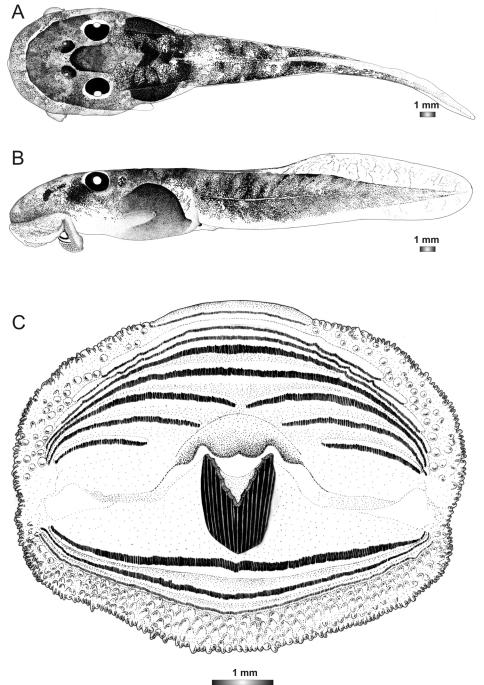
Drawings of the preserved DNA voucher tadpole of *Boophis sambirano* [Ca48](ZCMV 13109-ZSM 485/2010): **A** Dorsal view **B** Lateral view **C** Oral disc.

Morphological data were assessed in one tadpole (Figures 2 and 15) in developmental stage 27 (field number ZCMV 13109, ZSM 485/2010, BL 12.7 mm, TL 24.7 mm, Genbank accession number JQ518205) from Anjingo river (bridge at 57 km on the road from Antsohihy to Bealanana). The 16S rDNA sequence of this specimen was 94% identical to a reference sequence of *Boophis sambirano* tadpoles (accession EU717861) from Manongarivo Special Reserve. Two other voucher tadpoles are very similar to the described voucher specimen.

The tadpoles assigned to this candidate species have a similar oral disc as *Boophis sambirano* and *Boophis sambirano* [Ca47]. The higher number of marginal papillae (336) and of keratodonts on A_3_ (1052) differentiate these tadpoles from those of *Boophis sambirano* but are similar to *Boophis sambirano* [Ca47]. The ovoidal body form in dorsal view and the pigmentation pattern – variegated spots on the body and less coalesced spots on the tail musculature (Figure 1) – differentiate these tadpoles from those of *Boophis sambirano* [Ca47]. The beginning of the dorsal fin on the anterior 1/5 of the tail musculature is similar to that of *Boophis sambirano* [Ca47] but different from *Boophis sambirano*.

***Boophis sambirano* [Ca49 JQ518208*]***

**Figure 16.**
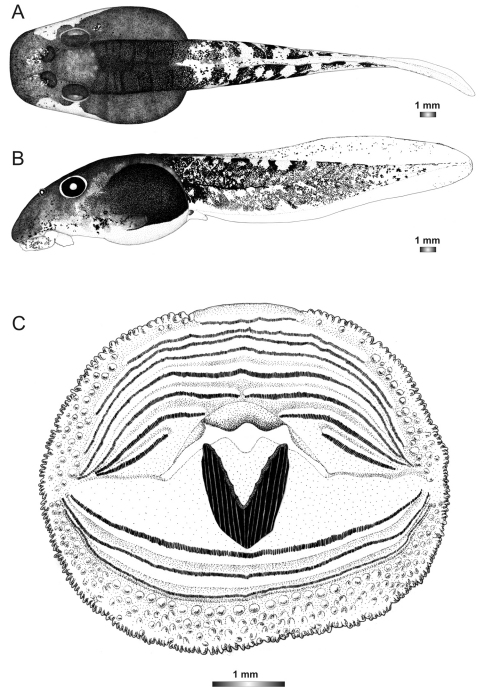
Drawings of the preserved DNA voucher tadpole of *Boophis sambirano* [Ca49](ZCMV 13155-ZSM 528/2010): **A** Dorsal view **B** Lateral view **C** Oral disc.

Morphological data were assessed in one tadpole (Figures 2 and 16) in developmental stage 27 (field number ZCMV 13155, ZSM 528/2010, BL 11.7 mm, TL 26.7 mm, Genbank accession number JQ518208) from Ankijagna Lagnana. The 16S rDNA sequence of this specimen is 94.1% identical to a reference sequence of *Boophis sambirano* tadpoles (accession EU717861) from Manongarivo Special Reserve. Three other voucher specimens and many non-voucher specimens of the same series are morphologically very similar to the described specimen.

The oral disc of the tadpoles assigned to this candidate species is the typical one of the *Boophis mandraka* group, characterized by a narrow dorsal gap of papillae (DG 23% of ODW) which is here wider than in *Boophis mandraka* [Ca46] but smaller than in the other tadpoles, and the short keratodont row A_1_ which is similar to that of *Boophis mandraka* [Ca46] tadpoles. The number of papillae is similar to that of *Boophis sambirano* and *Boophis mandraka* [Ca38]. These tadpoles can be easily distinguished from all *Boophis sambirano*-like tadpoles by their particular pigmentation pattern which is uniformly dark (Figure 1), by the non visibility of the lateral transparent area of the body wall, the ovoidal form of the body in dorsal view, and the eye position between the anterior 3/10 and 4/10 of the body.

***Boophis sambirano* [Ca50 JQ518211]**

**Figure 17.**
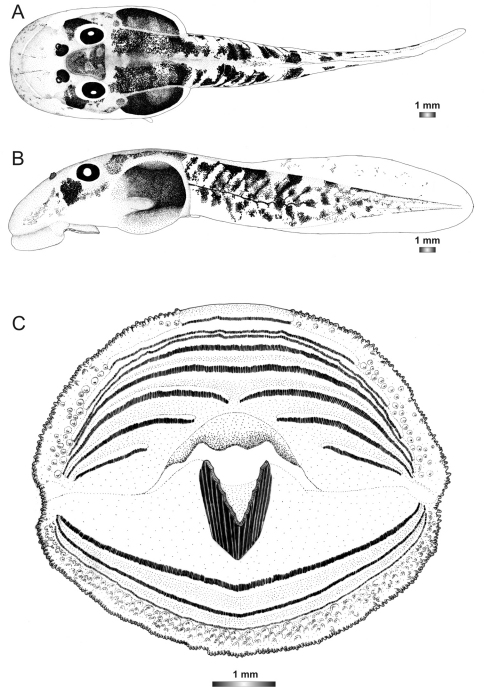
Drawings of the preserved DNA voucher tadpole of *Boophis sambirano* [Ca50](ZCMV 13172-ZSM 545/2010): **A** Dorsal view **B** Lateral view; **C** Oral disc.

Morphological data were assessed in one tadpole (Figures 2 and 17) in developmental stage 27 (field number ZCMV 13172, ZSM 545/2010, BL 11.7 mm, TL 25.7 mm, Genbank accession number JQ518211) from Ambinanitelo. The 16S rDNA sequence of this specimen is 94.9% identical to a reference sequence of *Boophis sambirano* tadpoles (accession EU717861) from Manongarivo Special Reserve. Three other voucher tadpoles are morphologically very similar to the described voucher specimen.

The oral disc of the tadpoles of this candidate species is similar to that of other *Boophis mandraka* group species. The tadpoles belonging to this candidate species have an elliptical body form in dorsal view but differ from those of *Boophis sambirano* [Ca49] by their pigmentation pattern. The presence of a lateral transparent area of the body wall surrounding the anterior 2/3 of the body is similar to those of *Boophis sambirano*, but the absence of contrasted integumental patches limiting the transparent body wall area surrounding the snout is a difference to *Boophis sambirano*, *Boophis sambirano* [Ca47], and *Boophis sambirano* [Ca48]. The tadpoles of this candidate species can thus be distinguished from those of other candidate species close to *Boophis sambirano* mainly by their coloration pattern (Figure 1).

### *Boophis majori* group

This group is heterogeneous in larval morphology and probably non monophyletic (e.g., Schmidt et al. 2008; Randrianiaina et al. 2009a). The rheophilous tadpoles in this group with an enlarged oral disc are further characterized by the absence of a lateral emargination, and absence of dorsal and ventral gaps of papillae. The submarginal papillae are complete. The anterior margin of the oral disc is separated by a deep crevice to the snout; i.e., the entire margin is free from the snout. LTRF 7(5–7)/3. The jaw sheaths are moderately strong and completely keratinized. The upper sheath always lacks a medial convexity. The lower sheath is U-shaped, ribbed, and higher than wide. Dorsolateral glands are present.

***Boophis marojezensis* Glaw & Vences, 1994**

**Figure 18.**
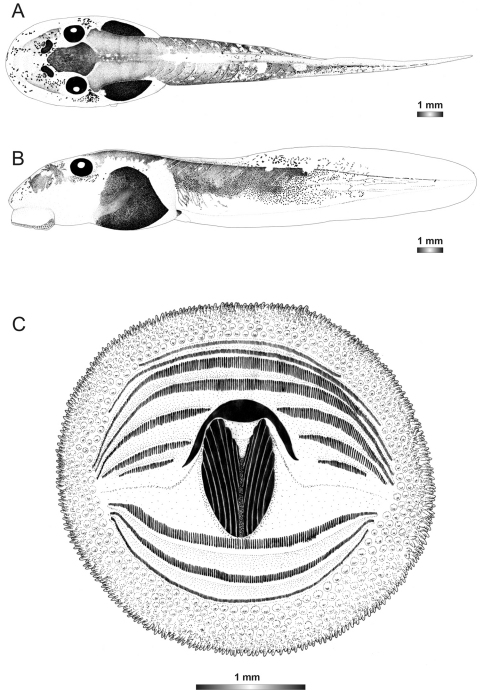
Drawings of the preserved DNA voucher tadpole of *Boophis marojezensis* (FGZC 2277-ZSM 1528/2007): **A** Dorsal view **B** Lateral view **C** Oral disc.

Morphological data were assessed in one tadpole (Figures 2 and 18) in developmental stage 27 (field number FGZC 2277, ZSM 1528/2007, BL 7.1 mm, TL 18.3 mm, Genbank accession number JQ518196), from Marojejy National Park. The 16S rDNA sequence of this specimen is 99.8% identical to a reference sequence of a *Boophis marojezensis* adult specimen (accession FJ559127) from the same locality. Three other voucher tadpoles are morphologically very similar to the described voucher specimen.

The tadpoles of this species are easily to distinguish from those belonging to other species groups (as described above) by the general structure of their oral disc which has no dorsal gap of papillae, and a LTRF of 7(5–7)/3. These tadpoles are also characterized by the highest number of submarginal papillae in *Boophis*, with 290 marginal and 606 submarginal papillae. The lateral transparent area of the body wall area is visible and the dorsolateral gland is obvious. The tail muscle is spotted and the spots fused to form patches mainly on the upper half of tail musculature, the density of the spots diminishes toward the tail tip. The posterior 1/3 of the tail has few pigments.

***Boophis marojezensis* [Ca25 Vieites et al. 2009]**

**Figure 19.**
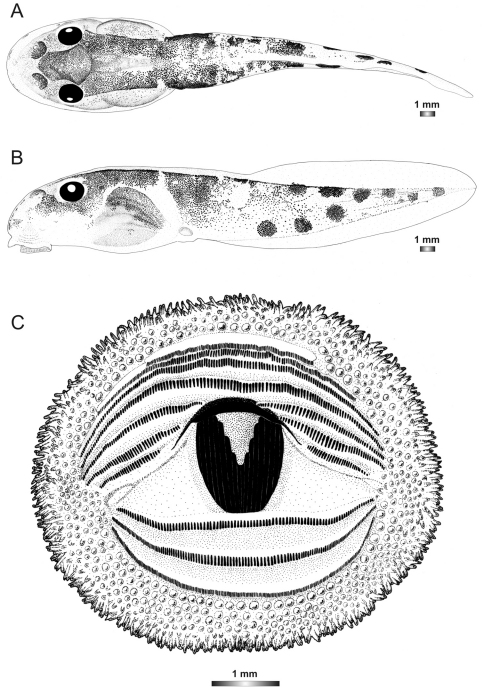
Drawings of the preserved DNA voucher tadpole of *Boophis marojezensis* [Ca25](FGZC 2929-ZSM 1611/2007): **A** Dorsal view **B** Lateral view **C** Oral disc.

Morphological data were assessed in one tadpole (Figures 2 and 19) in developmental stage 29 (field number FGZC 2929, ZSM 1611/2007, BL 7.8 mm, TL 18.5 mm, Genbank accession number FJ559146), from Marojejy National Park. The 16S rDNA sequence of this specimen is 97% identical to a reference sequence of *Boophis marojezensis* adult specimen (accession AY848596) from Vohidrazana, and less similar to other tadpoles from Marojejy. Two non-voucher specimens from the same series have the particular caudal pattern present in the voucher specimen.

Tadpoles assigned to this candidate species have the same oral disc structure as those of *Boophis marojezensis*, but with a lower number of papillae (222 marginal and 315 submarginal). The presence of seven more or less rounded patches formed by condensation of spots on the posterior half of the tail musculature of these tadpoles is a further useful character to differentiate them from those of *Boophis marojezensis*.

***Boophis marojezensis* [Ca26 Vieites et al. 2009]**

**Figure 20.**
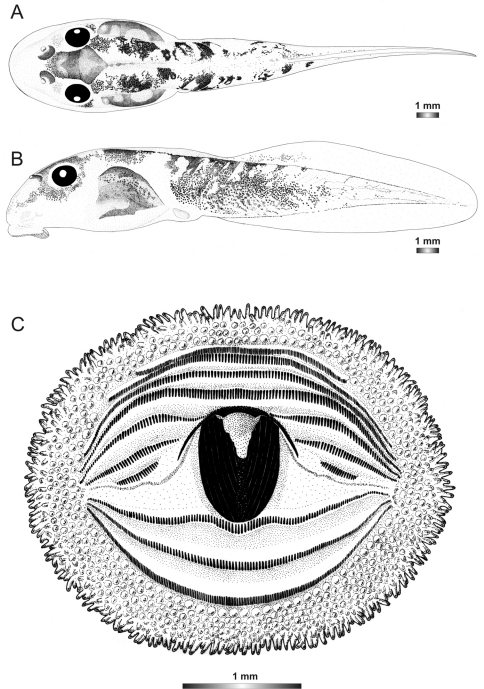
Drawings of the preserved DNA voucher tadpole of *Boophis marojezensis* [Ca26](FGZC 2930-ZSM 1612/2007): **A** Dorsal view **B** Lateral view **C** Oral disc.

Morphological data were assessed in one tadpole (Figures 2 and 20) in developmental stage 29 (field number FGZC 2930, ZSM 1612/2007, BL 8.8 mm, TL 20.6 mm, Genbank accession number JQ518197), from Marojejy National Park. The 16S rDNA sequence of this specimen is 96.6% identical to a reference sequence of a *Boophis marojezensis* adult specimen (accession AY848595) from Tsaratanana.

The single tadpole belonging to this candidate species has the typical *marojezensis*-like oral disc structure with 234 marginal and 430 submarginal papillae. It has almost the same pigmentation pattern as *Boophis marojezensis*, but the patches are more striking on the upper limit of tail musculature. It is differentiated from *Boophis marojezensis* [Ca25] by the absence of distinct patches on the tail musculature.

***Boophis marojezensis* [Ca51 JQ518198]**

**Figure 21.**
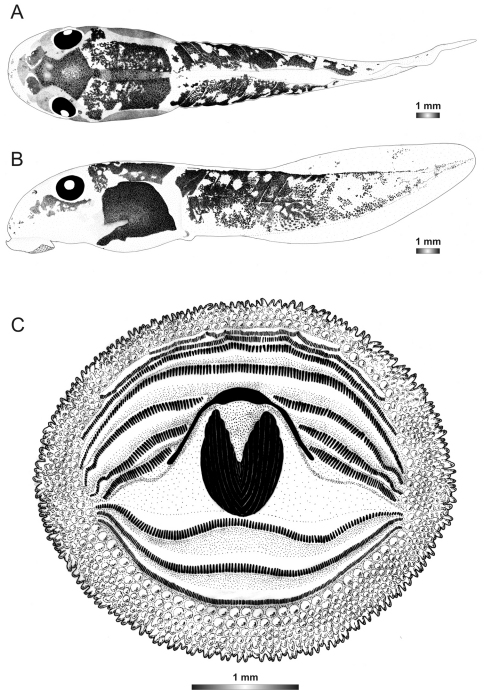
Drawings of the preserved DNA voucher tadpole of *Boophis marojezensis* [Ca51](ZCMV 3691-ZSM 267/2008): **A** Dorsal view **B** Lateral view **C** Oral disc.

Morphological data were assessed in one tadpole (Figures 2 and 21) in developmental stage 25 (field number ZCMV 3691, ZSM 267/2008, BL 6 mm, TL 20 mm, Genbank accession number JQ518198) from Ranomafana National Park. The 16S rDNA sequence of this specimen is 99.7% identical to a reference sequence of a *Boophis marojezensis* adult specimen (accession AY848594) from Vohiparara (but with >5% divergence to all other *Boophis marojezensis*-like forms). Twenty-one other tadpoles assigned to this candidate species reveal a similar morphological pattern and oral disc configuration as the described voucher specimen.

The tadpoles assigned to this candidate species have the typical *marojezensis*-like oral disc structure with 297 marginal and 309 submarginal papillae. They can be distinguished from the other *marojezensis*-like tadpoles by the absence of a lateral transparent area of the body wall area surrounding the body. They have also the widest inter-orbital distance (IOD) in the group, and they are also the only *marojezensis*-like tadpoles with eyes situated between the anterior 3/10 and 4/10 of the body. The tail muscle is covered by reticulations, mainly on the anterior half.

***Boophis marojezensis* [Ca52 JQ518215]**

**Figure 22.**
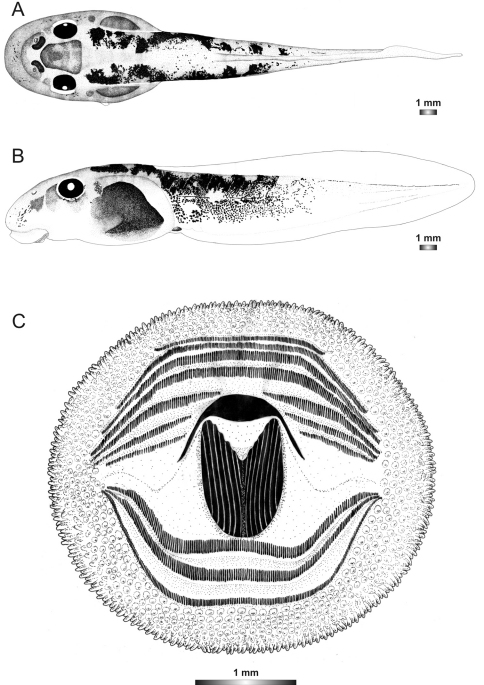
Drawings of the preserved DNA voucher tadpole of *Boophis marojezensis* [Ca52](ZCMV 13168-ZSM 541/2010): **A** Dorsal view **B** Lateral view **C** Oral disc.

Morphological data were assessed in one tadpole (Figures 2 and 22) in developmental stage 28 (field number ZCMV 13168, ZSM 541/2010, BL 10.5 mm, TL 26.1 mm, Genbank accession number JQ518215) from Ambinanitelo. The 16S rDNA sequence of this specimen is 100% identical to a reference sequence of anadult specimen assigned to *Boophis marojezensis* (accession AY848595) from Tsaratanana (but with >5% divergence to all other *Boophis marojezensis*-like forms). One other voucher specimen is morphologically very similar to the described one.

Tadpoles of this candidate species have the typical *marojezensis*-like oral disc structure with 258 marginal and 522 submarginal papillae. These tadpoles are distinguished from other *marojezensis*-like tadpoles by the only poorly recognizable lateral transparent body wall area surrounding the body, and by their tail pigmentation pattern which lacks melanophoric pigments (Figure 1). The position of the eyes is in the range of most other *Boophis marojezensis*-like tadpoles.

***Boophis marojezensis* [Ca53 JQ518216]**

**Figure 23.**
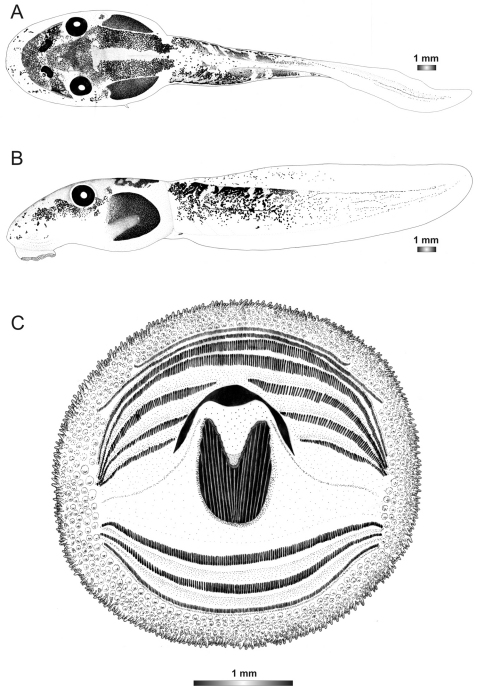
Drawings of the preserved DNA voucher tadpole of *Boophis marojezensis* [Ca53](ZCMV 13200-ZSM 573/2010): **A** Dorsal view **B** Lateral view **C** Oral disc.

Morphological data were assessed in one tadpole (Figures 2 and 23) in developmental stage 27 (field number ZCMV 13200, ZSM 573/2010, BL 9.6 mm, TL 23 mm, Genbank accession number JQ518216) from Tsaratanana Integral Reserve. The 16S rDNA sequence of this specimen is 98.8% identical to a reference sequence of a *Boophis marojezensis* adult specimen (accession FJ559127) from Marojejy. Five other voucher specimens attributed to the same candidate species are morphologically very similar to the described one.

The tadpoles of this candidate species have also a *marojezensis*-like oral disc with 243 marginal and 452 submarginal papillae. They are similar to *Boophis marojezensis*, *Boophis marojezensis* [Ca25], and *Boophis marojezensis* [Ca26], but different from *Boophis marojezensis* [Ca51] and *Boophis marojezensis* [Ca52] by the presence of a distinct lateral clear area surrounding the body (Figure 1). The general pigmentation pattern is similar to that of *Boophis marojezensis* [Ca26].

***Boophis vittatus* Glaw, Vences, Andreone & Vallan, 2001**

**Figure 24.**
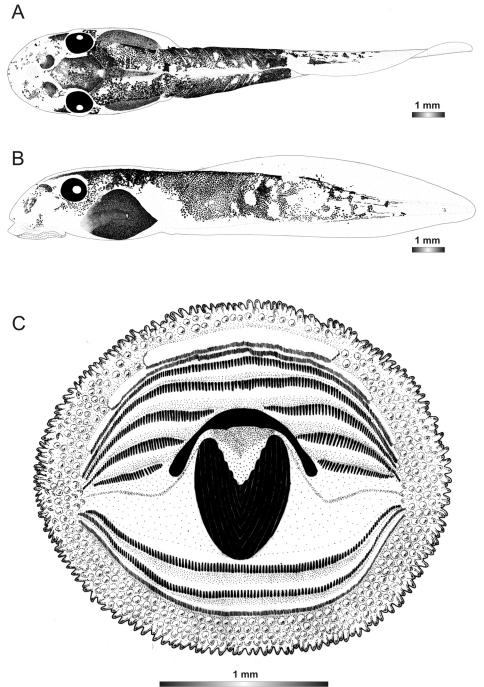
Drawings of the preserved DNA voucher tadpole of *Boophis vittatus* (FGZC 2238-ZSM 1906/2007): **A** Dorsal view **B** Lateral view **C** Oral disc.

Morphological data were assessed in one tadpole (Figures 2 and 24) in developmental stage 29 (field number FGZC 2238, ZSM 1906/2007, BL 7.8 mm, TL 18.5 mm, Genbank accession number JQ518200), from Marojejy National Park - Camp Mantella. The 16S rDNA sequence of this specimen is 100% identical to a reference sequence of a *Boophis vittatus* adult specimen (accession FJ559158) from the same locality. Three other voucher tadpoles of *Boophis vittatus* are morphologically very similar to the described voucher specimen.

The tadpoles of *Boophis vittatus* are the smallest tadpoles in this group. They have also a *marojezensis*-like oral disc structure with 289 marginal and 326 submarginal papillae. The tadpoles of this species are provided with a lateral transparent area of the body wall which is more pronounced surrounding the 2/3 anterior of the body. The tail musculature is reticulated like in *Boophis marojezensis* [Ca51].

### Occurrence of strongly rheophilous *Boophis* tadpoles in streams of Ranomafana

In streams of Ranomafana National Park, during the wet season, tadpoles of 44 frog species were found of which five had the morphological characteristics of the strongly rheophilous *Boophis*. These species were found in eleven out of 33 streams. *Boophis andohahela* occurred in eight streams with a mean of 9.9 specimens (min=1 to max=31 specimens), *Boophis ankaratra* occurred in two streams each with one single specimen, *Boophis marojezensis* [Ca51] was found in seven streams with a mean of 6.3 specimens (1 to 16 specimens), and only a single specimen of *Boophis schuboeae* was found. The tadpoles of *Boophis luciae* (named *Boophis* sp. 17 in Vieites et al. 2009) were found in eight streams with a mean of 12 specimens (1 to 33). During the dry season, 23 species were found of which three belong to the group of strongly rheophilous *Boophis*. Those species were found in 30% of the sampled streams in this season. *Boophis andohahela* occurred in 23% of the streams with nine specimens on average, *Boophis marojezensis* [Ca51] occurred in 30% of the sampled streams with three specimens on average, and *Boophis luciae* occurred in 15% of the sampled streams with eight specimens on average.

### Breeding site choice

**Figure 25.**
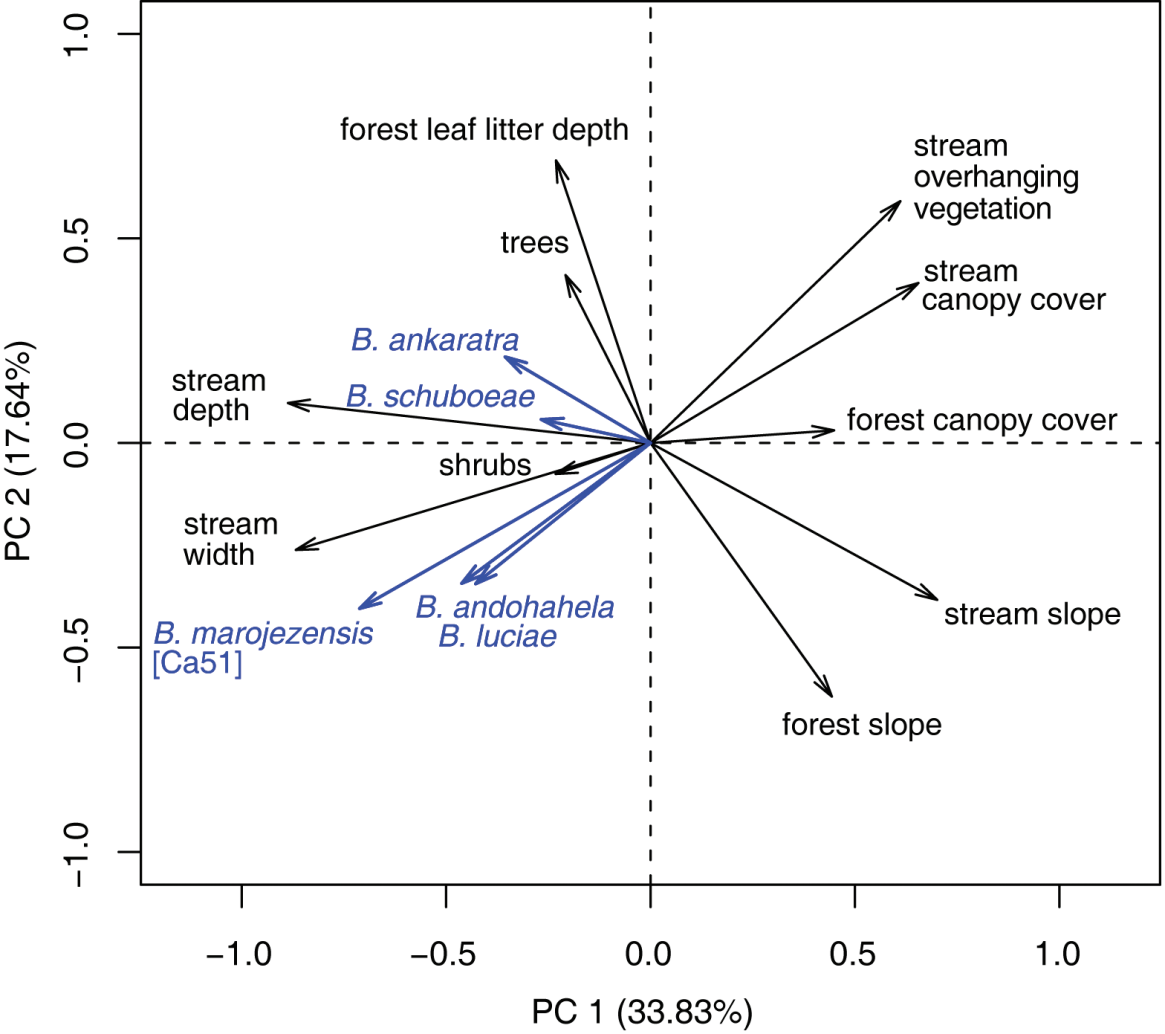
PCA biplot of variables of stream and surrounding habitat as recorded during a tadpole community study in Ranomafana National Park. The five present species of strongly rheophilous tadpoles are included as supplementary variables. Length and direction of vectors can be interpreted as correlations.

Principal Component Analysis on the habitat variables of the stream and the surrounding forest at Ranomafana resulted in three principal components, explaining together 65.5% of the variation in the data. We identified the following habitat variables being well represented (Figure 25): PC1 (33.8%) positive: slope and canopy cover of forest and stream, overhanging vegetation; negative: width and depth of the stream. Also four of the strongly rheophilous tadpole species, *Boophis ankaratra*, *Boophis andohahela*, *Boophis luciae*, and *Boophis marojezensis* [Ca51] are negatively correlated with this PC. The strongest contributors to PC2 (17.6%) were positive: forest leaf litter depth, stream overhanging plants, trees, and stream canopy cover; negative: slope of forest and stream. *Boophis andohahela* and *Boophis marojezensis* [Ca51] are negatively correlated with this PC. To PC3 (14.1%), the following variables were positive: number of small trees and shrubs in the forest and overhanging vegetation. Due to its rareness, no correlation of *Boophis schuboeae* incidence and PCs can be statistically assessed.

### Microhabitat choice

**Figure 26.**
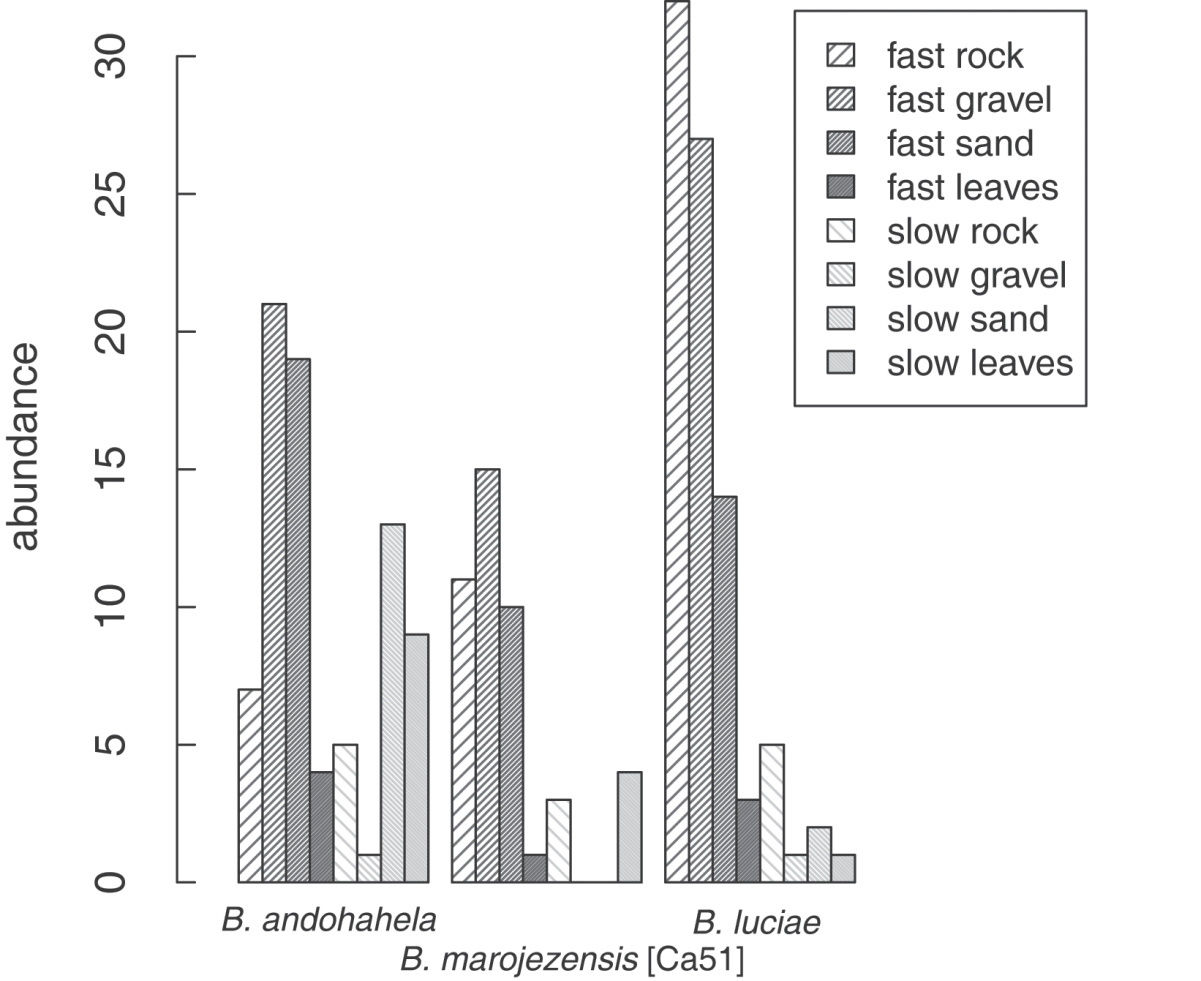
Tadpole distribution across the eight microhabitats (defined using water current and stream substrat) of the three most abundant strongly rheophilous *Boophis* that were sampled in Ranomafana National Park in wet season 2008. *Boophis andohahela*: N=8 , *Boophis marojezensis* [Ca51]: N=7, *Boophis luciae* N=10 with N= the number of streams.

Strongly rheophilous *Boophis* tadpoles were found in all microhabitats available in streams of Ranomafana National Park (Figure 26). A considerable amount of specimens was found in microhabitats characterised by fast flowing water and substrates of rock, gravel, and sand which generally do not harbour many tadpoles (own unpublished data). Tadpoles of *Boophis andohahela* were also relatively often found in slow moving parts of the streams with leaves and sand as substrates. Of the two locally rare species, *Boophis ankaratra* and *Boophis schuboeae*, one specimen of each was found in fast rock and fast sand microhabitat, and one specimen in slow rock microhabitat, respectively.

**Figure 27.**
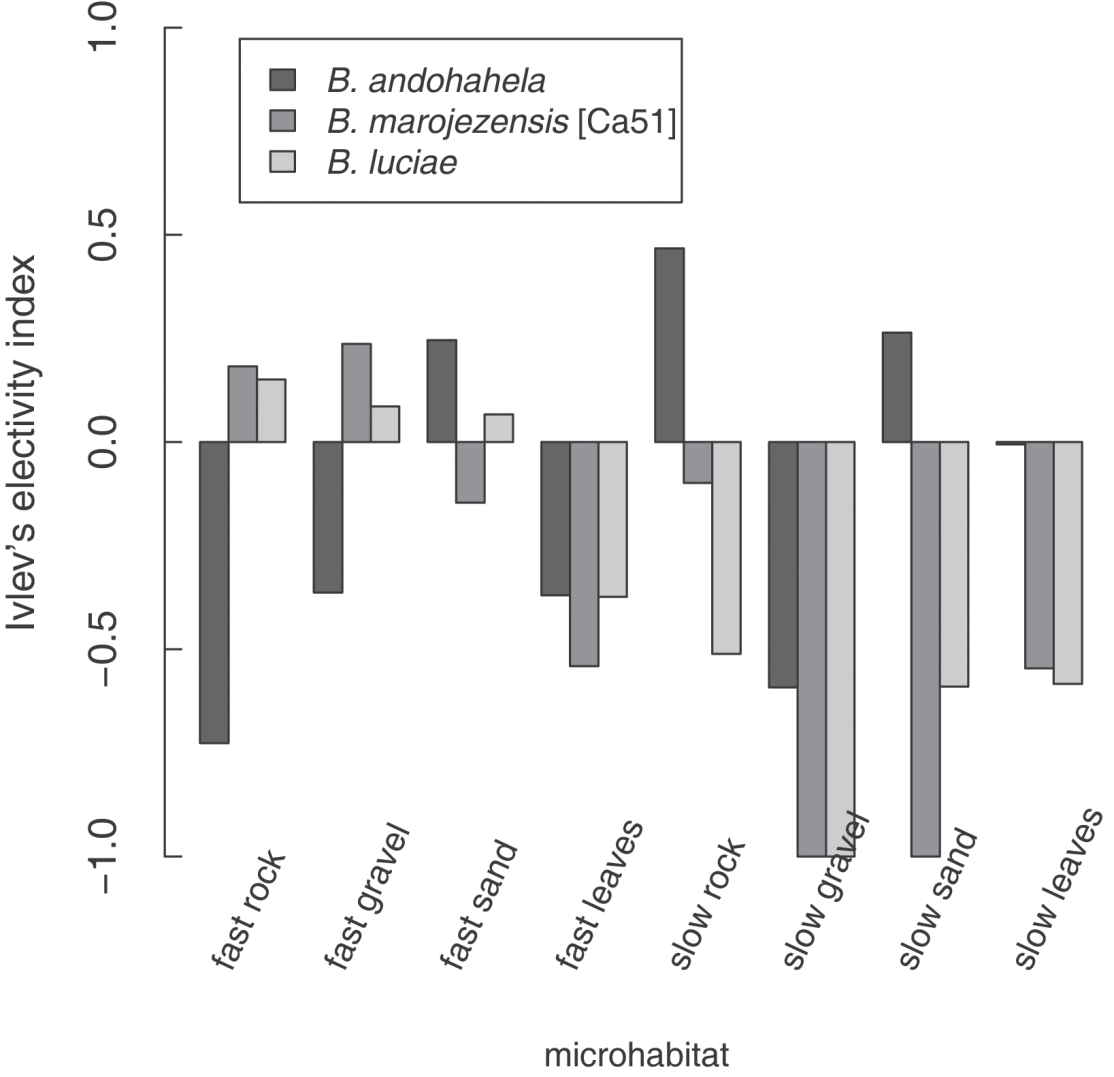
Barplot displaying tadpole microhabitat use of three most abundant *Boophis* species with strongly rheophilous tadpole morphology. Microhabitat use is calculated using Ivlev's electivity index (E, Ivlev 1961) with positive values representing microhabitat preferences and negative values representing microhabitat avoidance. For each species, only streams with at least eight specimens were used for analysis (*Boophis andohahela*: N=3, *Boophis marojezensis* [Ca51]: N=2, *Boophis luciae*: N=4 with N= the number of streams).

Considering the availability of microhabitats in the streams, Ivlev's electivity index (E, Ivlev 1961) shows that strongly rheophilous *Boophis* do not show a consistent microhabitat preference or avoidance except for “slow gravel” which is avoided by all species, and there is no general difference between the three species (Figure 27); factorial ANOVA without interaction term including only streams with at least eight specimens of the respective species; F_9,53_=1.716, p_model_=0.108, p_SG_=0.008, all other p including the factor “species” p>0.26). As interaction terms could not be included in this factorial ANOVA due to overparameterisation, we performed ANOVAs of subsets of the data and found that inter-species differences could only be shown for the microhabitat “fast rock” which is strongly avoided by *Boophis andohahela* (ANOVA of microhabitat subset; F_2,5_=22.6, p_model_=0.003, p*_B. andohahela_*<0.001) whereas *Boophis marojezensis* [Ca51] and *Boophis luciae* were found much more often than *Boophis andohahela* (p *_B. marojezensis_*_[Ca51]_=0.003, p*_B_*_. *luciae*_=0.002). For “slow sand”, only for *Boophis marojezensis* [Ca51] an avoidance could be detected (ANOVA of microhabitat subset; F_2,5_=3.829, p_model_=0.098, p*_B. marojezensis_*_[Ca51]_=0.047), *Boophis andohahela* and *Boophis luciae* used “slow sand” as much as available (p*_B. andohahela_*=0.427, p*_B_*_. *luciae*_=0.105). For all other microhabitats, no significant difference in microhabitat use of species could be detected. However, it should be noted that missing significances can be caused by the number of replicates (streams) which were reduced as we considered only streams with at least eight specimens of the respective species. A graphical evaluation of microhabitat use indicates that non-preferences or non-avoidances are in fact present (Figure 27).

## Discussion

### Comparisons to previous descriptions of strongly rheophilous *Boophis* tadpoles

Twenty-two strongly rheophilous tadpoles are characterized morphologically in this study, including fourteen tadpoles that are described for the first time and eight other species that had been previously described by other authors. Strongly rheophilous *Boophis* tadpoles have long been known by the work of Blommers-Schlösser (1979), and we here compare her descriptions of *Boophis majori*, *Boophis* sp., *Boophis erythrodactylus*, and *Boophis mandraka* larvae with current knowledge.

The tadpoles of *Boophis majori* described by Blommers-Schlösser (1979) correspond to those here assigned to the *Boophis marojezensis* complex, which is reasonable because also the taxonomic concept of *Boophis majori* of Blommers-Schlösser (1979) included *Boophis marojezensis*, a species that was only described later by Glaw et al. (2001). Assigning her *Boophis majori* tadpoles to one of the *Boophis marojezensis*-like candidate species is supported by their general external morphology with the sinistral spiracle situated on the 3/4 of the body, the well developed caudal musculature, the dark pattern on the body dorsum, the golden ventral side, the oral disc composed by complete small papillae, the lower jaw sheath that is higher than wide, the presence of an upper jaw sheath, and the LTRF of 7(5–7)/3. However, the finding of a dorsal gap of the papillae in some tadpoles is not in accordance with our description, because all *marojezensis*-like tadpoles (*Boophis marojezensis*, *Boophis marojezensis* [Ca25], *Boophis marojezensis* [Ca26], *Boophis marojezensis* [Ca51], *Boophis marojezensis* [Ca52], *Boophis marojezensis* [Ca53],and *Boophis vittatus*) lack a dorsal gap of papillae (data herein and in Raharivololoniaina et al. 2006). We conclude therefore that those tadpoles mentioned by Blommers-Schlösser (1979) might be strongly rheophilous tadpoles from another species, possibly belonging to the *Boophis albipuctatus* group. Additionally, the relative tail length which is two times of the body length and the situation of the nares close to the eyes of the tadpoles examined by Blommers-Schlösser (1979) do not agree with our data, because all relevant tadpoles in this study have a rather short tail (TAL 166 - 188% of BL) and an opening of the nares that is closer to the snout than to the eyes or in the middle (RN/NP 78 – 103%).

The tadpoles of *Boophis* sp. (Blommers-Schlösser, 1979) are similar to the *Boophis luteus* group tadpoles described herein according to their general oral disc structure. The LTRF 6(3–6)/3(1) corresponds to those of *Boophis englaenderi* tadpoles and 6(3–6)/3 to those of *Boophis englaenderi* [Ca23] tadpoles: This indicates that these tadpoles might belong to two different *Boophis* species. Since *Boophis englaenderi* and *Boophis englaenderi* [Ca23] do not occur in the site where Blommers-Schlösser (1979) observed her *Boophis* sp. tadpoles, we hypothesize that those tadpoles belong to species in *Boophis luteus* group whose larval stages are not yet known.

Tadpoles having narial openings closer to the eyes than to the snout, a sinistral spiracle situated on the 3/4 of the body, a well developed caudal musculature, a rounded oral disc with a LTRF of 7(5–7)/3, a dorsal gap of papillae and a complete jaw sheath were also described and assigned to *Boophis erythrodactylus*, a species of the *Boophis rappiodes* group, by Blommers-Schlösser (1979). The species identification of those tadpoles, however, is uncertain as already mentioned by Raharivololoniaina et al. (2006): *(1)* all the other species of the *Boophis rappiodes* group have generalized tadpoles, i.e., *Boophis rappiodes* as described in Blommers-Schlösser (1979) and Raharivololoniaina et al. (2006), *Boophis tasymena* and *Boophis viridis* in Raharivololoniaina et al. (2006), and *Boophis bottae* in Randrianiaina et al. (2009a), and *(2)* those tadpoles were stated to occur in sympatry with *Boophis mandraka* tadpoles, and either might just be a variation of *Boophis mandraka* tadpoles or belong to a closely related species of *Boophis mandraka* with similar tadpoles. As we encountered several times in our study, the tadpoles of two closely relative species can live sympatrically.

As described by Blommers-Schlösser (1979), the tadpoles of *Boophis mandraka* have a sinistral spiracle that opens at 2/3 of the body, narial openings closer to the eyes than to the snout, a tail that is two times longer than the body, a well developed caudal musculature, a silvery belly, an almost rounded oral disc with a V-shaped lower sheath and a LTRF of 7(6–7)/3. So far no strongly rheophilous tadpoles with only two interrupted upper keratodont rows have been observed in our study. We have observed in some tadpoles of *Boophis sibilans* and *Boophis luciae* that the gap separating the A_5_ row is very tight which might be responsible for the false impression of an uninterrupted row.

Tadpoles of *Boophis andohahela* from Ranomafana were described by Thomas et al. (2006). The general morphology and the oral disc structure of the tadpoles agrees with our specimens, except the keratodont row formula and the presence of a ventral gap of marginal papillae. Thomas et al. (2006) described tadpoles with a LTRF 6(3–6)/3(1), although in our study all tadpoles from the same locality as in Thomas et al. (2006) have a LTRF 6(3–6)/3. This might be caused by the fact that the teeth in the first lower row are very dense, and sometimes it folds in the middle giving the mistaking impression of a gap.

The *Boophis sibilans* tadpoles from Andasibe that Raharivololoniaina et al. (2006) described agree with our specimen except some minor differences; e.g., the relative width of the oral disc. These differences might be due to the different developmental stages of the tadpoles in the two studies, or by the different methods that have been used for taking the respective measurements.

Glos et al. (2007) described the tadpoles of *Boophis schuboeae* from Ranomafana and of *Boophis ankaratra* from Andringitra. The morphology therein is in accordance to the specimens of our study.

*Boophis englaenderi*, *Boophis vittatus* and *Boophis luciae* were described by Rasolonjatovo et al. (2010). We redescribe these species because of the bad condition of the voucher specimens and/or the lack of some data in the previous descriptions. The same tadpole specimen of *Boophis englaenderi* from Marojejy National Park was redescribed to facilitate the comparison to the other *Boophis luteus* group tadpoles. We furthermore described the tadpoles of *Boophis vittatus* and *Boophis luciae* from the same locality based on new voucher specimens because of the bad condition of the vouchers used in Rasolonjatovo et al. (2010).

### Morphological differences among tadpoles of closely related species

As decribed by Blommers-Schlösser (1979), defined by Raharivololoniaina et al. (2006), confirmed by Glos et al. (2007) and observed herein, strongly rheophilous tadpoles are typical stream-inhabiting organisms, and are characterized by a narrow and flat elongated body, a well developed caudal musculature, a wide oral disc with many small papillae that are either complete or interrupted by a dorsal gap, a rather small and ribbed (i.e., composed of a series of fused columns) lower jaw sheath, many upper keratodont rows with at least the two first being uninterrupted and three lower keratodont rows of which in most of the species the first one is uninterrupted.

This type of tadpoles can be found in different *Boophis* species groups: *Boophis luteus* group, *Boophis albipunctatus* group, *Boophis mandraka* group, and *Boophis majori* group (its occurrence in the *Boophis rappiodes* group is in need of confirmation). As described by Blommers-Schlösser (1979) and Schmidt et al. (2008), also *Boophis williamsi* (*Boophis microtympanum* group) has an enlarged oral disc (ODW 90% of BW, pers obs.) with a LTRF of 8(3–8)/3. However, we did not consider this species in our study because *(1)* this tadpole has a generalized oral disc structure (jaw sheaths, papillae and keratodonts) and *(2)* all the other strongly rheophilous tadpoles have a rather small size (BL 5.9 – 13.5 mm, TL 12.7 – 27.1 mm, in Gosner stages 25 – 36) compared to the montane *Boophis wiliamsi* tadpoles (BL 25.5 mm and TL 71.7 mm in Gosner stage 36).

Within the main groups of morphologically similar tadpoles, some can be very similar, but usually there are morphological details to differentiate them, whether in the external morphology or in the oral disc configuration; i.e., tadpoles that are very similar in external morphology can be differentiated in oral disc structure and vice versa:

(1) Three tadpoles belonging to the *Boophis luteus* group (*Boophis englaenderi*, *Boophis englaenderi* [Ca23], and *Boophis andohahela*) look alike in external morphology but can be differentiated easily by their keratodont row formula. Of these, *Boophis englaenderi* and *Boophis englaenderi* [Ca23] occur syntopically. The tadpoles of *Boophis englaenderi* [Ca23] can be distinguished from those of *Boophis englaenderi* by their relative tail length, by their pigmentation pattern and mainly by their oral disc structure (LTRF and number of papillae).

(2) In the *Boophis albipunctatus* group, *Boophis ankaratra*, *Boophis schuboeae*, *Boophis sibilans* and *Boophis luciae* are similar. *Boophis ankaratra* and *Boophis schuboeae* occur sympatrically, and they can be differentiated by the presence of dark pigmented bands on the tail muscle in *Boophis schuboeae*, and also by the absence of papillae on the lateral area where the oral disc folds in *Boophis schuboeae*. *Boophis sibilans* and *Boophis luciae* differ by the presence of a dark bridge which connects the dark sections on the tail muscle in *Boophis luciae*.

(3) All tadpoles known from the species of the *Boophis mandraka* group have a similar oral disc configuration, characterized by the absence of the upper jaw sheath and a LTRF of 8(6–8)/3. The tadpoles of *Boophis sambirano* and *Boophis mandraka* [Ca46] are very similar, except that *Boophis mandraka* [Ca46] has the narrowest dorsal gap of marginal papillae. The fact that these two tadpoles live allopatrically can help also to identify them. Five species of this group are distributed in close proximity in the North of Madagascar. Of these, *Boophis sambirano* [Ca47] and *Boophis sambirano* [Ca48] are sympatric, and can be differentiated by the patched *vs*. spotted pattern on the tail. *Boophis sambirano* [Ca49] and *Boophis sambirano* [Ca50] live also sympatrically. *Boophis sambirano* [Ca49] can be distinguished to the three other species by its generally dark coloration pattern, the ovoidal form of the body in dorsal view and the wide inter-orbital distance. *Boophis sambirano* [Ca50] can be differentiated by the intensity of the golden pigments which may cover the whole body and overlay the dark pigment in some specimens. *Boophis mandraka* [Ca38] is very typical by its weakly expressed pigmentation.

(4) Two tadpoles belonging to two different groups, *Boophis albipuncatus* (*Boophis albipunctatus* group) and *Boophis mandraka* [Ca38] (*Boophis mandraka* group) are similar in their weak expression of pigmentation, but they can easily differentiated by their oral disc morphology.

(5) Two cases of similarity are also found in *Boophis majori* group tadpoles. *Boophis marojezensis*, *Boophis marojezensis* [Ca26], *Boophis marojezensis* [Ca53] and *Boophis vittatus* are very similar in the presence of a clear, not pigmented lateral area surrounding the body, and in the tail pigmentation pattern. The fact that several of these species can occur sympatrically increases also the chance to confound them. On the other hand, the tadpoles of *Boophis marojezensis* [Ca51] and *Boophis marojezensis* [Ca52] are similar in the absence of a lateral clear area surrounding the body, and in their general pigmentation pattern. Only the tadpole of *Boophis marojezensis* [Ca25] is easily distinguishable by the presence of clear and more or less rounded patches on the tail muscle. As the three *Boophis marojezensis*-like tadpoles, *Boophis marojezensis*, *Boophis marojezensis* [Ca25] and *Boophis marojezensis* [Ca26], live syntopically in Marojejy National Park, *Boophis marojezensis* [Ca25] tadpoles will not be confounded with those of the two other species.

### Morphological clusters of strongly rheophilous *Boophis* tadpoles

Analyzing the structure of the oral disc of all these tadpoles allows classifying them into three clusters:

(1) The first cluster including three *Boophis luteus* group tadpoles is characterized by a moderately wide to very wide (ODW 56 to 84% of BW), non emarginated, ventrally positioned and oriented oral disc, which has an anterior margin connected directly to the snout, two uninterrupted upper rows of keratondonts (LTRF is 6(3–6)/3(1) for *Boophis englaenderi* but 6(3–6)/3 for the tadpoles of *Boophis englaenderi* [Ca23] and *Boophis andohahela*); a very long A_1_ (82 to 90% of ODW); a high number of keratodonts in A_1_ (220 to 301), totally keratinized; typically narrow to moderate sized jaw sheaths (JW 31 to 46% of ODW) with a very short medial convexity (MCL 0.04 to 0.11% of JW); a wide to very wide dorsal gap of papillae (DG 67 to 85% of BW); a low number of submarginal papillae (33 to 94) and a medium number of marginal papillae (101 to 175); a high positioned eye (EH 69 to 85% of BH) that is situated not far from midbody (SE 32 to 39% of BL); very high positioned nares (NH 57 to 82% of BH) that are situated below or at eye level (NH 82 to 97% EH) and closer to the snout than to the eye (RN 60 to 92% of NP); a short tail (TAL 155 to 183% of BL), and a developed caudal musculature.

(2) The second cluster is characterized by a wide to hyper-wide (ODW 74 to 108% of BW) non emarginated, ventrally positioned and oriented oral disc with an anterior margin separated from the snout by a shallow crevice or free; four or five uninterrupted upper rows of keratondonts giving a LTRF of 7(5–7)/3 or 8(6–8)/3); a short to moderately sized A_1_ (21 to 59% of ODW); a low to medium number of keratodonts in A_1_ (95 to 241), totally keratinized; U-shaped, ribbed narrow upper jaw sheaths (JW 30 to 34% of ODW which can present a small medial convexity or not); a U-shaped and "ribbed" lower sheath, a moderately wide to very narrow dorsal gap of papillae (DG 14 to 59% of BW); a medium to high number of marginal papillae (148 to 377), many submarginal papillae (190 to 368); high to very high positioned eyes (EH 71 to 84% of BH) that are situated closer to the snout than to midbody (SE 35 to 49% of BL), high to very high positioned nares (NH 64 to 92% of BH) that are situated below or above the eye level (NH 86 to 112% of EH) and closer to the eye than to the snout (RN 107 to 194% of NP); a short to very short tail (TAL 146 to 184% of BL); and a developed caudal musculature. Tadpoles of the *Boophis albipuncatus* group (*Boophis schuboeae*, *Boophis ankaratra*, *Boophis albipunctatus*, *Boophis sibilans*, and *Boophis luciae*) and *Boophis mandraka* group (*Boophis sambirano*, *Boophis mandraka* [Ca38], *Boophis mandraka* [Ca46], *Boophis sambirano* [Ca47], *Boophis sambirano* [Ca48], *Boophis sambirano* [Ca49], and *Boophis sambirano* [Ca50]) belong to this cluster. All *Boophis mandraka* group tadpoles lack a keratinized upper jaw sheath.

(3) The third cluster is characterized by a wide (ODW 68 to 79% of BW) non emarginated, ventrally positioned and oriented oral disc without a dorsal gap of papillae and with the anterior margin being free from the snout; four uninterrupted upper keratodont rows (LTRF 7(5–7)/3); a moderately sized A_1_ (45 to 52% of ODW); a medium number of keratodonts in A_1_ (126 to 235); a totally keratinized upper jaw sheath (JW 30 to 38% of ODW) without medial convexity; a U-shaped and ribbed lower sheath, many submarginal (222 to 318) and marginal (206 to 522) papillae; high positioned eyes (EH 68 to 80% of BH) that are situated closer to midbody (SE 35 to 49% of BL); very high positioned nares (NH 68 to 80% of BH) that are situated below eye level except for *Boophis vittatus* and *Boophis marojezensis* [Ca25] (NH 89 to 101% of EH) and closer to the snout for the most (RN 78 to 109 % of NP); a very short to short tail (TAL 140 to 188% of BL), and a developed caudal musculature. Tadpoles of the *Boophis majori* group (*Boophis marojezensis*, *Boophis marojezensis* [Ca25], *Boophis marojezensis* [Ca26], *Boophis marojezensis* [Ca51], *Boophis marojezensis* [Ca52], *Boophis marojezensis* [Ca53], and *Boophis vittatus*) belong to this cluster.

Although we lack at this time an explicit and well supported phylogenetic hypothesis for the relationships among all these species of *Boophis*, the morphological characters of these three morphological clusters can serve to develop a possible evolutionary scenario of the origin of the specializations in strongly rheophilous *Boophis* tadpoles, departing from the structures in more generalized *Boophis* tadpoles. All of the latter are characterized by having one (the first) uninterrupted upper keratodont row and one (the first) interrupted lower keratodont row, typically smooth (non-ribbed) jaw sheaths and a medial convexity in the upper jaw sheath (see Raharivololoniaina et al. 2006, Randrianiaina et al. 2009a, b, and Rasolonjatovo et al. 2010). Specialization to a strongly rheophilous life thus involves *(1)* reduction of the size of the jaw sheaths correlated with *(2)* the disappearance of the medial convexity, *(3)* reduction of the size of the dorsal gap of marginal papillae, *(4)* reduction of the length of the row A_1_, *(5)* reduction of the number of keratodonts in A_1_, compensated by an increase of the number of *(6)* marginal and *(7)* submarginal papillae and *(8)* of the uninterrupted upper keratodont rows.

The decrease of the size of the jaw sheaths may provoke the fading of its medial convexity on one hand and leaves a place for many dorsal and lateral, even ventral submarginal papillae, and new uninterrupted upper keratodont rows on the other hand. Also, the reduction of the size of the dorsal gap leads to a higher number of marginal papillae. The development of many dorsal marginal papillae reduces the area available for the first upper keratodont row and thus may cause the reduction of its length, which in turn leads to the decrease of the number of the teeth. However, the loss of the upper jaw sheath in all species and candidate species of the *Boophis sambirano* complex is still unclear. This characteristic is neither caused by a fixation artifact nor by the transportation of the specimens because we observed it already in the living tadpoles in the field (Figure 29), and because it is consistent within series. The absence of the upper jaw sheath was found even in young tadpoles (Gosner 25) indicating that it occurs very early in larval development. It remains to be tested (e.g., by a study on embryonic development), however, if this structure never develops, or is initially formed but then disappears at some early developmental stage.

### Ecomorphological guilds in *Boophis* tadpoles

A magnitude of descriptions of the larval stages of Madagascan frogs have been recently published (Andreone et al. 2002, Glos and Linsenmair 2005, Raharivololoniaina et al. 2003, 2006, Thomas et al. 2005, 2006, Altig and McDiarmid 2006, Vejarano et al. 2006a, b, c, Glos et al. 2007, Schmidt et al. 2008, 2009a, b, Grosjean and Vences 2009, Jovanovic et al. 2009, Rasolonjatovo et al. 2010, Grosjean et al. 2006, 2007, 2011a, b, Randrianiaina et al. 2007, 2009a, b, 2011a, b, c). While some of them merely intended to increase general knowledge on Madagascan tadpoles, others attempted to classify the tadpoles into ecomorphological guilds. For *Boophis* tadpoles, Raharivoloniaina et al. (2006) tried to define three guilds, named A,Band C, mainly based on three variables: relative width of oral disc, number of inframarginal papillae, and number of keratodonts on the first anterior row. As already mentioned by Randrianiaina et al. (2009a), these guilds were not intended to replace nor to refine the guilds of Altig and Johnston (1989), but to achieve a complementary, more quantitative classification that would better fit the variation of the *Boophis* tadpoles studied. Moreover, the criteria chosen by Rahavololoniaina et al. (2006) were few and some of those that Altig and Johnston (1989) used do not exist in *Boophis* tadpoles (Randrianiaina et al. 2009a). Therefore, a comprehensive definition of adequate guilds for Malagasy tadpoles will require the consideration of numerous new variables without omitting those that have been used before. In this process it is important to notice first the presence or absence of one component (e.g., jaw sheath and keratondont) and then its configuration (e.g., totally or poorly keratinized sheaths, density of papillae; Randrianiaina et al. 2011a).

According to Altig and Johnston (1989), three different guilds might correspond to *Boophis* tadpoles. The clasping tadpoles have a dorsal gap of marginal papillae, commonly five keratodont rows (but as numerous as 8/8), usually with anterior rows that are more numerous than posterior rows (e.g., 9/3), and a globular to slightly depressed body. They inhabit medium to slow water currents and the maintenance of their position in the water current with the help of the oral disc is of minor importance. The adherent tadpoles have small and complete marginal papillae, and a LTRF of commonly 2/3. They inhabit faster flowing water than clasping tadpoles, their position maintenance via the oral disc is common to continuous, and their body is often depressed. The suctorial tadpoles have a depressed body, small and complete marginal papillae, and a LTRF from 2/3 to a maximum of 17/21. They inhabit even faster running waters than the clasping and adherent tadpoles, and their position maintenance via their oral disc is continuous.

In this study, no new guild names are defined, but we suggest to adapt in a preliminary way the guilds already defined by Altig and Johnston (1989).

(1) We do not consider the *Boophis luteus* group tadpoles truely strongly rheophilous, due to their more generalized and intermediate characteristics. These tadpoles (*Boophis englaenderi*, *Boophis englaenderi* [Ca23], *Boophis andohahela*) can possible be considered to be part of the “clasping” guild.

(2) The first guild of strongly rheophilous tadpoles, here considered as “adherent”, is the second category of tadpoles classified in the previous section which is composed by the tadpoles of the *Boophis albipuncatus* group (*Boophis schuboeae*, *Boophis ankaratra*, *Boophis albipunctatus*, *Boophis sibilans*, and *Boophis luciae*) and the *Boophis mandraka* group (*Boophis sambirano*, *Boophis mandraka* [Ca38], *Boophis mandraka* [Ca46], *Boophis sambirano* [Ca47], *Boophis sambirano* [Ca48], *Boophis sambirano* [Ca49], and *Boophis sambirano* [Ca50]), because they inhabit faster running water and the maintenance of the position in the water via their oral disc is common to continuous. This guild is characterized mainly by the presence of a dorsal gap of papillae and two typical LTRF-s which are 8(5–8)/3 and 8(6–8)/3. All *Boophis mandraka* group tadpoles lack an upper jaw sheath, while this structure is present in the *Boophis albipuncatus* group tadpoles.

(3) The second guild that we define as “suctorial” is the third category of tadpoles classified in the previous section which is composed of all *Boophis marojezensis*-like tadpoles (*Boophis marojezensis*, *Boophis marojezensis* [Ca25], *Boophis marojezensis* [Ca26], *Boophis marojezensis* [Ca51], *Boophis marojezensis* [Ca52], *Boophis marojezensis* [Ca53], and *Boophis vittatus*). They probably inhabit faster running water and maintain continuously their position in the water with the help of their oral disc because of the complete state of the papillae that they have. This guild is characterized by the absence of a dorsal gap of papillae and a LTRF of 7(5–7)/3.

### Habitat selection and ecology of strongly rheophilous *Boophis* tadpoles

**Figure 28.**
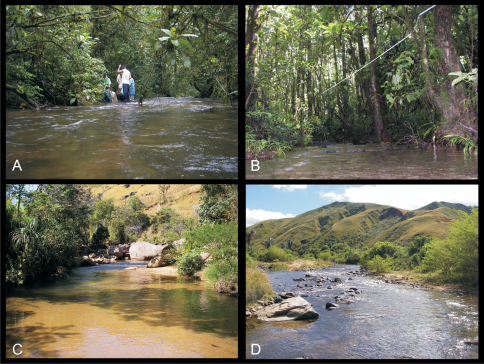
Pictures showing tadpole capture sites inside a primary forest in Ranomafana National Park (**A** in Fompohonina river, **B** in Piste E 100 stream), and outside the forest (**C** in Anjingo river and **D** in Ankijagna Lagnana).

In the tropical rainforest of Ranomafana National Park, strongly rheophilous *Boophis* tadpoles occur throughout the whole year (own unpublished data) with clearly higher abundances in the wet season. Whereas some species are relatively common (e.g., *Boophis marojezensis* and *Boophis luciae*), others are locally extremely rare (e.g., *Boophis ankaratra*, *Boophis schuboeae*). In this area, strongly rheophilous *Boophis* do neither include the most common tadpoles species nor is the group itself as common as other groups (Grosjean et al. 2011). Species of this group choose larger, open, slowly running streams for breeding (Figure 28); small streams with high slope and a dense vegetation cover are generally avoided. This is generally true for all strongly rheophilous species studied in Ranomafana National Park. The latter kind of stream might be avoided as they are less attractive to adults than large streams, which provide more space without the risk of egg and tadpole predation by fishes. Small streams might also be characterised by reduced food availability, e.g., due to reduced periphyton growth as a result of high vegetation coverage (Mallory and Richardson 2005; Altig et al. 2007). This actually describes the expected pattern for most tadpoles in Madagascan rainforest streams and can also be observed, e.g., for tadpoles of the *Mantidactylus* subgenus *Ochthomantis*, which are characterised by reduced oral disc structures (Randrianiaina et al. 2011a). In contrast, the also specialized funnel mouthed tadpoles of *Mantidactylus* subgenus *Chonomantis* do not follow this pattern, as for some species no prediction of occurrence by habitat characteristics is possible and some species (e.g., *Mantidactylus opiparis*) prefer combinations of habitat characteristics that are unfavourably represented in our PCs (Grosjean et al. 2011a).

Within the streams, however, strongly rheophilous *Boophis* tadpoles are quite outstanding regarding their microhabitat choice compared to other abundant and well observed tadpole groups. This is especially true for two of the most common of these species, *Boophis marojezensis* [Ca51] and *Boophis luciae*, and less pronounced for *Boophis andohahela*, consistent with the more generalized oral disc structure of this latter species. Whereas we could not show true preferences for fast running sections, we could at least show that a considerable number of specimens are indeed using these faster parts of the streams. This clearly separates these tadpoles from other abundant groups (Grosjean et al. 2011a, Randrianiaina et al. 2011a), and most likely reflects the morphological specialisations of oral disc, body, and tail to withstand the current.

**Figure 29.**
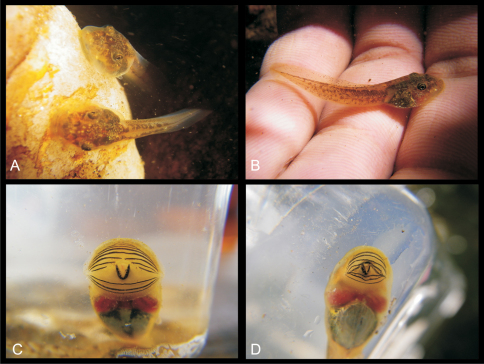
Photographs in life of strongly rheophilous *Boophis* tadpoles: **A** and **B** Underwater pictures of *Boophis sambirano*-like tadpoles (from Antevialambazaha - Tsaratanana Integral Reserve) **C** and **D** Oral disc of *Boophis sambirano* [Ca48] (ZCMV 13109 -ZSM 485/2010) and *Boophis marojezensis* [Ca52](ZCMV 13169-ZSM 542/2010) (from Ambinanitelo) fixing on the sides of an aquarium.

Their large ventral oral disc allows attaching on substrate (Figure 29) such as rocks and gravel, and the presence of numerous short papillae presumably aids in forming a tight seal between the oral disc and the irregularities of substrate (Altig and McDiarmid 1999). Also, their relatively small body size and well developed caudal musculature probably allows a good locomotory performance in strong current.

### Reverse taxonomy and high cryptic species diversity of *Boophis*

As already demonstrated by Randrianiaina et al. (2011a), reverse taxonomy, initially defined for unicellular organisms and invertebrates, can also be applied to better studied groups such as vertebrates. Herein we confirm the usefulness of this method by finding numerous divergent tadpole DNA sequences. Twelve candidate species are defined in this study by the divergent DNA sequence of the tadpoles in comparison with the sequences of all species and candidate species previously known by adult specimens. To evaluate the status of such genetically divergent specimens, it is important to evaluate whether (1) the genetic divergence is correlated with other characters, e.g., consistent morphological differences, and (2) whether these consistently differentiated groups may furthermore occur in sympatry, which then suggests they are reproductively isolated evolutionary lineages, and thus, distinct species. We could indeed find such a situation in three pairs of species, and thus can flag several of the newly discovered genealogical lineages as confirmed candidate species (Vieites et al. 2009):

(1) *Boophis englaenderi* [Ca23] lives syntopically with *Boophis englaenderi*, and these two forms show clear and constant differences genetically and in larval morphology, as described above, including characters of the oral disc, relative tail length, and coloration.

(2) In the *Boophis mandraka* group, *Boophis sambirano* [Ca49] tadpoles are very deviant and can easily be differentiated by coloration and the position of the eyes from the lineage *Boophis sambirano* [Ca50] occurring at a nearby locality in the same stream.

(3) *Boophis marojezensis* [Ca25] is very distinct by the presence of more or less rounded patches on the posterior half of the tail musculature which distinguishes it from the two syntopic forms, *Boophis marojezensis* and *Boophis marojezensis* [Ca26].

As a conclusion, this extraordinary and surprising diversity of *Boophis marojezensis*-like and *Boophis sambirano*-like candidate species especially in northern Madagascar probably indeed reflects a high number of yet undescribed species, and claims for a biogeographic and evolutionary explanation. It further confirms that stream-breeding frogs apparently show a higher geographical structuring of their diversity (e.g., Inger et al. 1974; Vences et al. 2002). An in-depth revision of these frogs is necessary to understand this diversity and its taxonomic relevance, and needs to be based on an integrative approach assessing their bioacoustic, and nuclear genetic divergence, focusing on sympatric occurrences which we expect to be particularly informative regarding the isolation mechanisms between these lineages.

## Appendix

### Appendix 1

Table 3–5. (doi: 10.3897/zookeys.178.1410.app1 File format: Excel spreadsheet (xls).

**Explanation note:** Appendix I contains Table 3–5.

### Appendix 2

Morphological description of the rheophilous *Boophis* tadpoles. (doi: 10.3897/zookeys.178.1410.app2) File format: MS Word document (.doc).

**Explanation note:** Appendix II contains complete morphological description of the rheophilous *Boophis* tadpoles.

## References

[B1] Altig R Johnston GF (1989) Guilds of anuran larvae: Relationships among developmental modes, morphologies, and habitats. Herpetological Monographs 3: 81-109. doi: 10.2307/1466987

[B2] Altig R McDiarmid RW (1999) Body plan: Development and morphology. In: McDiarmid RW Altig R (Eds). Tadpoles: the Biology of Anuran Larvae. Chicago University Press, New York: 24-51.

[B3] Altig R McDiarmid RW (2006) Descriptions and biological notes on three unusual mantellid tadpoles (Amphibia: Anura: Mantellidae) from southeastern Madagascar. Proceedings of the Biological Society of Washington 119: 418-425. doi: 10.2988/0006-324X(2006)119[418:DABNOT]2.0.CO;2

[B4] Altig R Whiles MR Taylor CL (2007) What do tadpoles really eat? Assessing the trophic status of an understudied and imperilled group of consumers in freshwater habitats. Freshwater Biology 52: 386-395. doi: 10.1111/j.1365-2427.2006.01694.x

[B5] Andreone F Vences M Guarino FM Glaw F Randrianirina JE (2002) Natural history and larval morphology of *Boophis occidentalis* (Anura: Mantellidae: Boophinae) provide new insights into the phylogeny and adaptive radiation of endemic Malagasy frogs. Journal of Zoology 257: 425-438. doi: 10.1017/S0952836902001036

[B6] Blommers-Schlösser R (1979) Biosystematics of the Malagasy frogs. II. The genus *Boophis* (Rhacophoridae). Bijdragen tot de Dierkunde 49: 261-312.

[B7] Box GEP Cox DR (1964) SeriesB(Methodological) 26: 211-252.

[B8] Crawley MJ (2007) The R Book. John Wiley & Sons Ltd, West Sussex, England. doi: 10.1002/9780470515075

[B9] Dubois A (1995) Keratodont formulae in anuran tadpoles: Proposal for standardisation. Journal of Zoological Systematics and Evolutionary Research 33: 1-15.

[B10] Fox J (2008) CAR: Companion to applied regression, R Package version 1.2–16. Online at http://cran.r-project.org/web/packages/car/index.html

[B11] Glaw F Köhler J de la Riva I Vieites DR Vences M (2010). Integrative taxonomy of Malagasy treefrogs: combination of molecular genetics, bioacoustics and comparative morphology reveals twelve additional species of *Boophis*. Zootaxa 2383: 1-82.

[B12] Glaw F Vences M (2007) A Field Guide to the Amphibians and Reptiles of Madagascar, 3^rd^ edition. Vences and Glaw Verlag, Köln, 496.

[B13] Glaw F Vences M Andreone F Vallan D (2001) Revision of the *Boophis majori* group (Amphibia: Mantellidae) from Madagascar, with descriptions of five new species. Zoological Journal of the Linnean Society 133: 495–529. doi; 10.1111/j.1096-3642.2001.tb00637.x

[B14] Glos J Linsenmair KE (2005) Description of the tadpoles of *Boophis doulioti* and *B. xerophilus* from Western Madagascar with notes on larval life history and breeding ecology. Amphibia-Reptilia 26: 459-466. doi: 10.1163/156853805774806287

[B15] Glos J Teschke M Vences M (2007) Aquatic zebras? The tadpoles of the Madagascan treefrogs *Boophis schuboeae* Glaw & Vences 2002 compared to those of *Boophis ankaratra* Andreone 1993. Tropical Zoology 20: 125-133.

[B16] Gosner KL (1960) A simplified table for staging anuran embryos and larvae with notes on identification. Herpetologica 16: 183-190.

[B17] Grosjean S Vences M (2009) The tadpole of the toadlet *Scaphiophryne marmorata* from Madagascar. Zootaxa 1986: 67-68.

[B18] Grosjean S Thomas M Glaw F Vences M (2006) The tadpole of the Malagasy treefrog *Boophis rufioculis*: molecular identification and description. Spixiana 29: 73-76.

[B19] Grosjean S Glos J Teschke M Glaw F Vences M (2007) Comparative larval morphology of Madagascan toadlets of the genus *Scaphiophryne*: phylogenetic and taxonomic inferences. Zoological Journal of the Linnean Society 151: 555-576. doi: 10.1111/j.1096-3642.2007.00329.x

[B20] Grosjean S Strauß A Glos J Randrianiaina RD Ohler A Vences M (2011a) Morphological uniformity in the funnel-mouthed tadpoles of Malagasy litter frogs, subgenus *Chonomantis*. Zoological Journal of the Linnean Society 162: 149-183.

[B21] Grosjean S Randrianiaina RD Strauß A Vences M (2011b) Sand-eating tadpoles in Madagascar: morphology and ecology of the unique larvae of the treefrog *Boophis picturatus*. Salamandra 47 (2): 75-88.

[B22] Inger RF Voris HK Voris HH (1974) Genetic variation and population ecology of some Southeast Asian frogs of the genera *Bufo* and *Rana*. Biochemical Genetics 12: 121-145. doi: 10.1007/BF00487821 4424654

[B23] Ivlev VS (1961) Experimental Ecology of the Feeding of Fishes. Yale University Press, New Haven, 320 pp.

[B24] Jovanovic O Glos J Glaw F Randrianiaina RD Vences M (2009) Comparative larval morphology in Madagascan frogs of the genus *Mantella* (Amphibia: Mantellidae). Zootaxa 2124: 21-37.

[B25] Lê S Josse J Husson F (2008) FactoMineR: An R Package for Multivariate Analysis.

[B26] Mallory MA Richardson JS (2005) Complex interactions of light, nutrients and consumer density in a stream periphyton-grazer (tailed frog tadpoles) system. Journal of Animal Ecology 74: 1020-1028. doi: 10.1111/j.1365-2656.2005.01000.x

[B27] Padial JM Miralles A de la Riva I Vences M (2010) The integrative future of taxonomy. Frontiers in Zoology 7: 16-16. doi: 10.1186/1742-9994-7-16 20500846 PMC2890416

[B28] Palumbi SR Martin A Romano S McMillian WO Stine L Grabowski G (1991) The simple fools guide to PCR, v.2.0. Department Zoology, Kewalo Marine Laboratory, University of Hawaii, Honolulu.

[B29] Raharivololoniaina L Vieites DR Glaw F Vences M (2003) Larval stages, habitat and distribution of the hyperoliid frog *Heterixalus rutenbergi* (Boettger 1881). Alytes 21(1–2): 59-65.

[B30] Raharivololoniaina L Grosjean S Raminosoa N Glaw F Vences M (2006) Molecular identification, description and phylogenetic implications of the tadpoles of 11 species of Malagasy treefrogs, genus *Boophis*. Journal of Natural History 40: 1449-1480. doi: 10.1080/00222930600902399

[B31] Randrianiaina RD Glaw F Thomas M Glos J Raminosoa N Vences M (2007) Descriptions of the tadpoles of two species of *Gephyromantis*, with a discussion of the phylogenetic origin of direct development in mantellid frogs. Zootaxa 1401: 53-61.

[B32] Randrianiaina RD Raharivololoniaina L Preuss C Strauß A Glaw F Teschke M Glos J Raminosoa N Vences M (2009a) Descriptions of the tadpoles of seven species of Malagasy treefrogs, genus *Boophis*. Zootaxa 2021: 23-41.

[B33] Randrianiaina RD Navarro Antúnez R Canitz J Forth F Lemme I Rodríguez B Rinas H Thänert, R Tröger P Westphal N Willim A Wollenberg KC Strauß A Vences M (2009b) Vogue or adaptive character? A tadpole's goatee helps to distinguish two cryptic treefrog species of the genus *Boophis*. Herpetology Notes 2: 165-173.

[B34] Randrianiaina RD Strauß A Glos J Glaw F Vences M (2011a) Diversity, external morphology and “reverse taxonomy” in the specialized tadpoles of Malagasy river bank frogs of the subgenus *Ochthomantis* (genus *Mantidactylus*). Contributions to Zoology 80 (1): 17-65.

[B35] Randrianiaina RD Köhler J Glos J Vences M Glaw F (2011b) Where to grow in the Tsingy? Limestone rock pools as breeding habitats of the relict frog *Tsingymantis antitra* from Madagascar and description of its tadpole. Salamandra 47 (2): 77-89.

[B36] Randrianiaina RD Wollenberg KC Rasolonjatovo Hiobiarilanto T Strauß A Glos J Vences M (2011c) Nidicolous tadpoles rather than direct development in Malagasy frogs of the genus *Gephyromantis*. Journal of Natural History 5: 2871-2900. doi: 10.1080/00222933.2011.596952

[B37] Rasolonjatovo Hiobiarilanto T Randrianiaina RD Glos J Strauß A Vences M (2010) Description of ten tadpoles in the genus *Boophis* from Madagascar. Zootaxa 2694: 1-25.

[B38] R Development Core Team (2009) R: A language and environment for statistical computing. R Foundation for Statistical Computing, Vienna, Austria.

[B39] Schmidt H Strauß A Reeve E Letz A Ludewig A-K Neb D Pluschzick R Randrianiaina RD Reckwell D Schröder S Wesolowski A Vences M (2008) Descriptions of the remarkable tadpoles of three treefrog species, genus *Boophis*, from Madagascar. Herpetology Notes 1: 49-57.

[B40] Schmidt H Strauß A Glaw F Teschke M Vences M (2009a) Description of tadpoles of five frog species in the subgenus *Brygoomantis* from Madagascar (Mantellidae: Mantidactylus). Zootaxa 1988: 48-60.

[B41] Schmidt H Glaw F Teschke M Vences M (2009b) The tadpole of the Madagascar bullfrog, *Laliostoma labrosum*. Zootaxa 2005: 67-68.

[B42] Strauß A Reeve E Randrianiaina RD Vences M Glos J (2010) The world's richest tadpole communities show functional redundancy and low functional diversity: ecological data on Madagascar's stream-dwelling amphibian larvae. BMC Ecology 10: 12-12. 20459864 10.1186/1472-6785-10-12 PMC2877654

[B43] Thomas M Raharivololoniaina L Glaw F Vences M Vieites DR (2005) Montane tadpoles in Madagascar: molecular identification and description of the larval stages of *Mantidactylus elegans*, *Mantidactylus madecassus*, and *Boophis laurenti* from the Andringitra Massif. Copeia 2005: 174-183. doi: 10.1643/CH-03-293R2

[B44] Thomas M Raharivololoniaina L Glaw F Vences M (2006) Description of the tadpole of the Malagasy treefrog *Boophis andohahela*. Alytes 23: 96-102.

[B45] Vallan D Vences M Glaw F (2010) Forceps delivery of a new treefrog species of the genus *Boophis* from eastern Madagascar (Amphibia: Mantellidae). Amphibia-Reptilia 31: 1-8. doi: 156853810790457830

[B46] Vejarano S Thomas M Vences M (2006a) Comparative larval morphology in Madagascan frogs of the genus *Guibemantis* (Anura: Mantellidae). Zootaxa 1329: 39-57.

[B47] Vejarano S Thomas M Glaw F Vences M (2006b) Advertisement call and tadpole morphology of the clutch-guarding frog *Mantidactylus argenteus* from eastern Madagascar. African Zoology 41: 164-169. doi: 10.3377/1562-7020(2006)41[164:ACATMO]2.0.CO;2

[B48] Vejarano S Thomas M Vences M (2006c) Comparative tadpole morphology in three species of frogs of the genus *Spinomantis* (Amphibia: Mantellidae). Contributions to Zoology 75: 99-108.

[B49] Vences M Andreone F Glaw F Kosuch J Meyer A Schaefer H-C Veith M (2002) Exploring the potential of life-history key innovation: brook breeding in the radiation of the Malagasy treefrog genus *Boophis*. Molecular Ecology 11: 1453-1463. doi: 10.1046/j.1365-294X.2002.01543.x 12144665

[B50] Vences M Thomas M Bonett RM Vieites DR (2005) Deciphering amphibian diversity through DNA barcoding: chances and challenges. Philosophical Transactions of the Royal Society B: Biological Sciences 360: 1859-1868. doi: 10.1098/rstb.2005.1717 PMC1609216 16221604

[B51] Vences M Andreone F Glos J Glaw F (2010a) Molecular and bioacoustic differentiation of *Boophis occidentalis* with description of a new treefrog from north-western Madagascar. Zootaxa 2544: 54-68.

[B52] Vences M Köhler J Crottini A Glaw F (2010b) High mitochondrial sequence divergence meets morphological and bioacoustic conservatism: *Boophis quasiboehmei* sp. n., a new cryptic treefrog species from south-eastern Madagascar. Bonn Zoological Bulletin 57: 241-255.

[B53] Vieites DR Wollenberg KC Andreone F Köhler J Glaw F Vences M (2009) Vast underestimation of Madagascar's biodiversity evidenced by an integrative amphibian inventory. Proceedings of the National Academy of Sciences of the United States of America 106: 8267-8272. doi: 10.1073/pnas.0810821106 19416818 PMC2688882

[B54] Zuur AF Ieno-Graham EN Smith GM (2007) Analysing Ecological Data. Springer Science + Business Media, New York, 672 pp.

